# *Candida auris* cells form giant lipid droplets to survive in harsh environments

**DOI:** 10.1038/s42003-025-08204-7

**Published:** 2025-05-22

**Authors:** Qiushi Zheng, Chao Li, Tianren Hu, Jian Bing, Clarissa J. Nobile, Haiqing Chu, Guanghua Huang

**Affiliations:** 1 State Key Laboratory of Genetic Engineering, School of Life Sciences, Shanghai Institute of Infectious Disease and Biosecurity, and Department of infectious diseases, Huashan Hospital, Fudan University, Shanghai, China; 2 Department of Respiratory and Critical Care Medicine, Shanghai Pulmonary Hospital, School of Medicine, Tongji University, Shanghai, China; 3 Shanghai Key Laboratory of Tuberculosis, Shanghai Pulmonary Hospital, School of Medicine, Tongji University, Shanghai, China; 4 Department of Molecular and Cell Biology, University of California, Merced, CA USA; 5 Health Sciences Research Institute, University of California, Merced, CA USA; 6 College of Pharmaceutical Sciences, Southwest University, Chongqing, China

**Keywords:** Fungi, Pathogens

## Abstract

The emerging fungal pathogen *Candida auris* is notorious for environmental persistence, which is a major contributor to outbreaks in healthcare settings. Here we report that giant lipid droplets (gLDs) inside *C. auris* cells play critical roles in the ability to survive harsh environments. *C. auris* cells that contain gLDs exhibit an increased tolerance to environmental stresses, antifungals, and host-associated antimicrobial peptides. These cells often undergo significant cell wall remodeling and sloughing of the outer layer of the cell wall. Lipidomics analysis indicates that cells with gLDs contain a significantly higher level of triacylglycerols, sterol esters, and other lipids, such as glycerolipids, sphingolipids, and sterol lipids. At the transcriptional level, a large set of differentially expressed genes was observed between *C. auris* cells with and without gLDs. Our study reveals that gLDs are a new strategy used by *C. auris* to adapt to stressful conditions and to persist in hospital environments.

## Abstract (web summary)

The superbug *Candida auris* is able to form giant lipid droplets to survive in harsh environment, which may contribute to hospital outbreaks.

## Introduction

Nosocomial pathogens often use multiple strategies to adapt to hostile environments^1,2^. This adaptation is critical for the pathogens to cause host infections, survive in the hospital environment, and effectively transmit between the environment and humans or from humans to humans. Understanding the underlying adaptive mechanisms of pathogens in response to environmental fluctuations will help in the development of new strategies to control associated infections and hospital outbreaks.

First described in 2009 in Japan^3^, the emerging and drug-resistant fungal pathogen *Candida auris* has been considered to be a serious global public health problem^4–6^. This fungal pathogen rapidly spread worldwide over the last decade and has been identified in more than 50 countries on six continents as of 2023^6,7^. Bloodstream infections caused by *C. auris* often lead to high patient mortality rates^8,9^. In addition, *C. auris* is able to colonize and survive on human skin and environmental surfaces for extended periods of time. This ability to persist in the environment has been thought to be a major contributor to its rapid inter-hospital and intra-hospital transmission rates as well as its likelihood to cause outbreaks^6,7^. Moreover, *C. auris* is highly tolerant to disinfectants, desiccation, high temperatures, and high-saline environments^10–12^, further contributing to its environmental persistence.

Thermotolerance is an important property of *C. auris* that is not only essential for its survival and replication in mammalian hosts but is also critical for its pathogenesis^13^. It is hypothesized that thermal adaptation by fungi is associated with climate change^14,15^. An overall increase in environmental temperatures could drive the evolution of thermotolerance in *C. auris* within its natural environmental niches, where it was then disseminated by an intermediate host such as sea birds^13^. In previous publications, we and others found that *C. auris* could grow at high temperatures ( ≥ 40 °C) in medium containing 10% NaCl (wt/vol)^16,17^. These characteristics of elevated thermal and salinity tolerance could make *C. auris* a potent nosocomial pathogen and contribute to its persistence in the environment and its transmission within hospital settings. Similar to other pathogenic *Candida* species, in response to environmental changes, *C. auris* is able to undergo morphological transitions and form biofilms^4,18^. Several morphological phenotypes such as yeast-form, hyphal, and multicellular aggregates have been observed in *C. auris*^19–22^. Different cell types often exhibit different abilities to survive in stressful conditions.

Despite the considerable attention that *C. auris* has received from both basic research and clinical communities over the past decade, the underlying mechanisms of this pathogen’s unique capabilities of long-term environmental persistence, transmissibility, and survival in the host remain unclear. Lipid droplets (LDs) are ubiquitous subcellular organelles, which not only serve as lipid reservoirs for cellular physiology but also play critical roles in stress responses in both single-celled and multicellular organisms^23–26^. Previous studies on the other fungal species also indicate that LDs are involved in the regulation of cell wall remodeling and spore formation in fungi under stressful conditions^27,28^. In this study, we report that *C. auris* cells form giant lipid droplets (gLDs) to survive harsh environments both in vitro and in vivo. Compared to control cells, *C. auris* cells containing gLDs exhibit an obviously enhanced ability to persist on the surfaces of medical materials and in the presence of environmental and antifungal stresses. *C. auris* cells with gLDs also exhibit an increased ability to colonize host tissues. Key genes involved in triacylglycerol (TAG) and sterol esters (SE) synthesis, the major components of lipid droplets, and mitochondrial metabolism are required for the formation of gLDs and survival of *C. auris* cells under stressful conditions. Taken together, the formation of gLDs could be a novel strategy used by *C. auris* to adapt to environmental stresses and contribute to its transmissibility and outbreak potential in hospital settings.

## Results

### *C. auris* cells form giant lipid droplets (gLDs) on nutrient-poor media

**Fig. 1:**
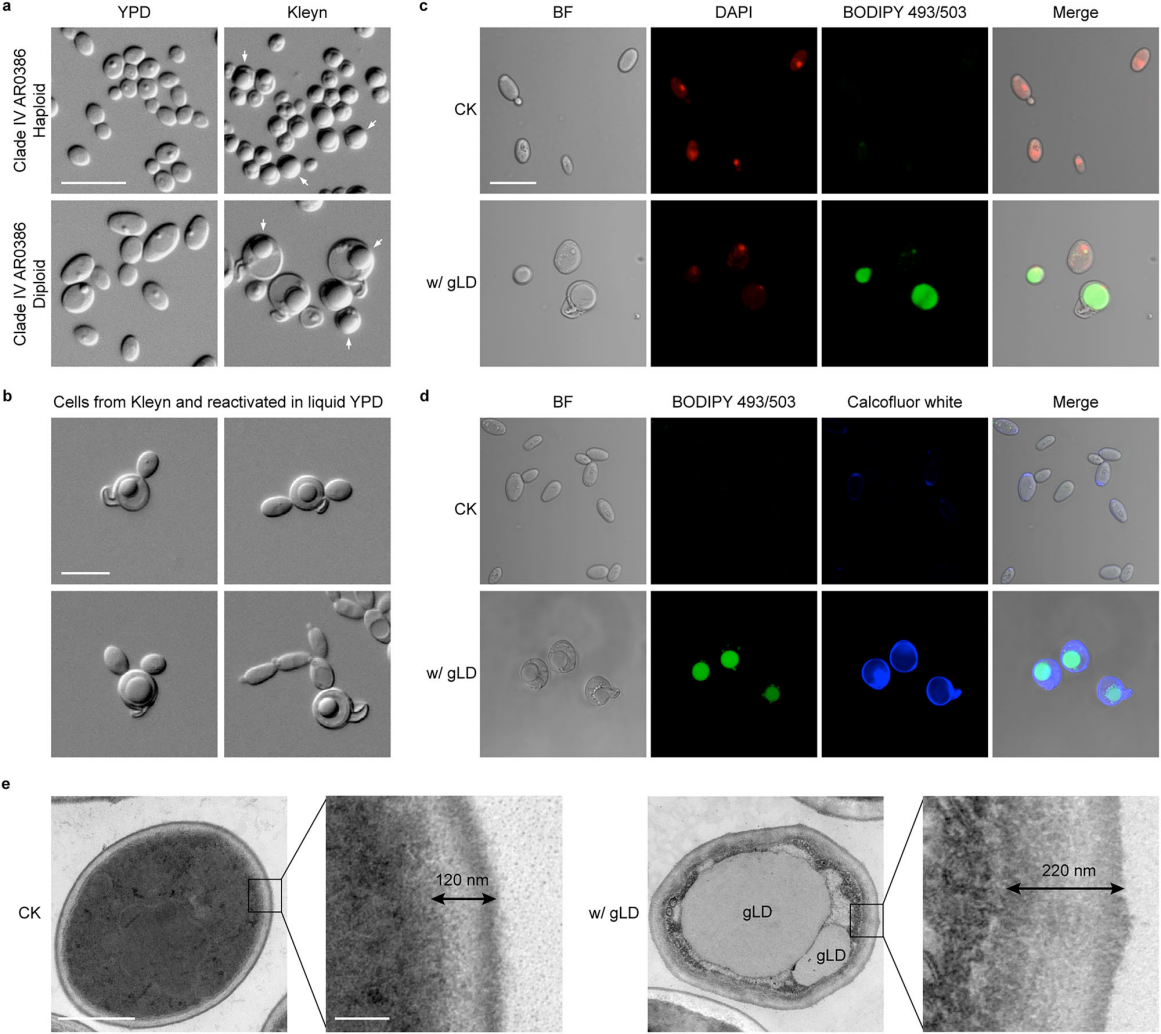
Cellular morphologies of *C. auris* cells with giant lipid droplets (gLDs). *C. auris* strain AR0386 (Clade IV) was examined. **a** Cellular morphologies of haploid and diploid cells on nutrient-rich YPD and nutrient-poor Kleyn media. Differential interference contrast (DIC) microscopy assays were performed. *C. auris* cells were initially grown on YPD medium for 2 days at 30 °C. Then, 5 × 10^6^ cells in 10 μL ddH_2_O were spotted onto YPD or Kleyn medium and incubated at 30 °C for 7 days. Arrows indicate cells with gLDs. Percentage of cells with gLDs for the haploid strain on Kleyn medium: 53.4 ± 4.6%; for the diploid strain: 61.7 ± 1.7%. Percentage of cells with projections for the haploid strain on Kleyn medium: 0.4 ± 0.3%; for the diploid strain: 6.6 ± 1.5%. No gLDs and cell wall projections were observed on YPD medium for both the haploid and diploid strains. **b** Morphologies of *C. auris* diploid cells with gLDs re-grown in fresh liquid YPD medium for 3 h at 37 °C. Retention of the lipid droplets in the mother cells was observed. **c** DAPI and BODIPY 493/503 staining assays for genomic DNA and lipid droplets, respectively. **d** Calcofluor white and BODIPY 493/503 staining assays for cell wall chitin and lipid droplets, respectively. For panels (**a**–**d**), scale bar, 10 μm. **e** Transmission electron microscopy (TEM) images of *C. auris* cells with gLDs (right) or without gLDs (left, CK). The size of the cell with two gLDs: 2.4 μm × 3.0 μm in diameter; the size of the big gLD: 1.6 μm × 2.0 μm in diameter; the size of the small gLD: 0.4 μm × 0.6 μm in diameter. Cell wall thicknesses are marked. BF brightfield. Scale bar for cells, 1 μm; scale bar for cell wall structures, 0.1 μm. For panels (**c**–**e**), diploid cells of strain AR0386 grown on Kleyn medium were examined.

Although *C. auris* has traditionally been considered to be a haploid species, it can undergo spontaneous switching between haploid and diploid cell states under certain in vitro culture conditions and during systemic infections^29^. We set out to investigate whether *C. auris* diploid cells could undergo meiosis or sporulation when cultured on Kleyn medium, a standard nutrient-poor medium used for *Saccharomyces cerevisiae* sporulation assays^30^. Haploid *C. auris* cells served as a reference control. When cultured on Kleyn medium plates at 30 °C for 7 days, one or multiple spore-like subcellular organelles were formed in both haploid and diploid cells (Fig. 1a). This phenomenon, however, was not observed when *C. auris* cells were cultured on YPD rich medium. Interestingly, *C. auris* cells with spore-like subcellular organelles (especially in the case of diploid cells) often had a projected structure when cultured on Kleyn medium (Fig. 1a, b). We then collected the *C. auris* cells containing spore-like organelles and regrew them in fresh liquid YPD medium. We found that these cells underwent normal cell divisions and produced daughter cells (Fig. 1b). The spore-like organelles were retained in the mother cells, indicating that they were not “endospores” or “sexual spores”. Schaeffer-Fulton staining (negative for spores, not shown) and DAPI staining (for genomic DNA) assays confirmed this finding (Fig. 1c).

We next examined whether the spore-like structures were lipid droplets (LDs) by staining the *C. auris* cells with BODIPY 493/503, a conventional lipid dye. Strong signals were observed in the cells containing spore-like structures by fluorescence microscopy (Fig. 1c, d), indicating that these cellular organelles were LDs. Transmission electron microscopy (TEM) assays verified that they were in fact giant lipid droplets (gLDs) within *C. auris* cells (Fig. 1e). Interestingly, TEM assays demonstrated that *C. auris* cells containing gLDs often had a thickened cell wall structure (Fig. 1e). Calcofluor white staining assays demonstrated that the thickened cell wall contained a remarkably increased level of chitin (Fig. 1d). Quantitative assays confirmed the change of cell wall composition (Supplementary Table 1).

We next investigated whether the formation of gLDs was a general feature of *C. auris* clinical strains. Haploid and diploid cells of strains BJCA001 (clade I), CBS 12373 (clade II), RICU2 (clade III), and AR0386 (clade IV) were grown on Kleyn medium to support the development of gLDs. Both haploid and diploid cells of all strains tested were able to form gLDs (Supplementary Fig. 1), suggesting that this phenomenon is a general feature of *C. auris* clinical isolates of different genetic clades. Moreover, we found that when cultured on water agar medium (ddH_2_O + 4% agar, representing an extremely nutrient-poor condition and mimicking hospital surfaces), both haploid and diploid *C. auris* cells formed gLDs (Supplementary Fig. 2). Taken together, our findings indicate that nutrient stress promotes the development of gLDs in *C. auris*.

### *C. auris* cells with gLDs undergo cell wall remodeling and sloughing of the outer layer

Since a portion of *C. auris* cells with gLDs had a projected structure (Fig. 1a, b), we observed the cell walls of these cells in more detail by scanning electron microscopy (SEM) and TEM. SEM and TEM assays demonstrated that detached cell wall structures were present on the cells containing gLDs (Supplementary Fig. 3), indicating that the cell wall was undergoing a form of remodeling and/or rebuilding concomitant with the formation of gLDs. It remains to be investigated whether these detached cell wall structures are involved in cell survival or immune responses during *C. auris* colonization or infection.

### *C. auris* cells form giant lipid droplets (gLDs) on the surfaces of medical supplies

A striking feature of *C. auris* is the ability to survive and persist in hospital environments and on the surface of medical materials and devices for extended periods of time^6,7^. We next tested whether *C. auris* could form gLDs on medical supplies, thereby increasing its persistence. *C. auris* cells were inoculated on the surfaces of several kinds of medical supplies and incubated at 30 °C for 14 days. Both haploid and diploid cells of strain AR0386 formed obvious gLDs on the surfaces of all medical supplies tested (Supplementary Fig. 4), which included masks (polypropylene), gloves (butyronitrile), tapes (cotton), silica gel, culture dishes (polystyrene), and coverslips (borosilicate glass). These findings indicate that the formation of gLDs within *C. auris* cells could be a common phenomenon in healthcare settings and a potential adaptive fungal response mechanism to environmental stressors.

### Sodium acetate promotes the formation of gLDs in *C. auris* under in vitro culture conditions

To reveal the mechanisms of gLD formation in *C. auris*, we next attempted to figure out the factors that induce gLD formation under harsh growth conditions. The components of Kleyn medium include glucose, peptone, NaCl, and sodium acetate (NaAc)^30^. Acetate can promote lipid synthesis through acetyl-CoA and the tricarboxylic acid (TCA) cycle^31^. To examine whether NaAc is required for the formation of gLDs, we grew *C. auris* cells on Kleyn medium (w/ NaAc), Kleyn medium w/o NaAc, and modified YPD medium (1/10 YPD and 1/10 YPD w/ NaAc). Both haploid and diploid cells of *C. auris* formed clear larger LDs on the media containing NaAc than on the media lacking NaAc (Supplementary Fig. 5). Interestingly, we found that sodium dihydrogen phosphate (NaH_2_PO_4_) had a strong inhibitory effect on the formation of gLDs in *C. auris* (Supplementary Fig. 5). The addition of NaH_2_PO_4_ to the media lacking NaAc prevented LD formation in both *C. auris* haploid and diploid cells (Supplementary Fig. 5). Based on these findings and hereafter Kleyn medium (w/ NaAc) and modified Kleyn medium (mKleyn, w/o NaAc and w/ NaH_2_PO_4_) were used as our assays for the induction and repression of gLDs, respectively.

### *C. auris* cells form gLDs in a mouse outer ear infection model

**Fig. 2:**
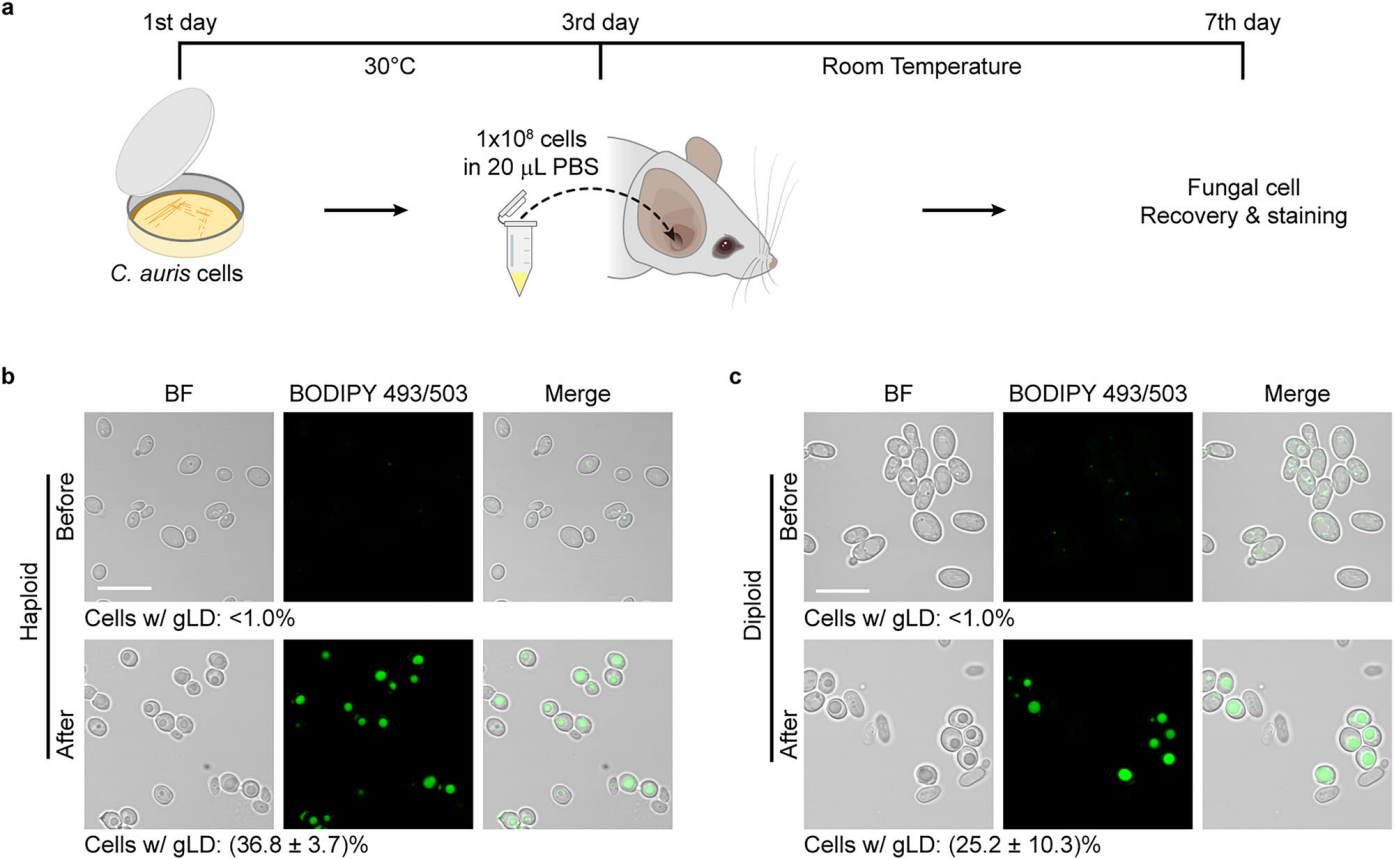
Formation of gLDs in *C. auris* in a mouse ear infection model. **a** Schematic diagram of the *C. auris* infection assay using a mouse ear model. Haploid and diploid cells of strain AR0386 were grown on YPD medium for 2 days at 30 °C. *C. auris* cells were collected, washed and suspended in PBS. 20 μL suspension containing 1 × 10^8^ *C. auris* cells were inoculated in the mouse ear canal. After 5 days of infection, *C. auris* cells were collected from the mouse auricle for staining assays. **b**, **c** Morphologies of *C. auris* haploid or diploid cells before inoculation (upper panel) and cells collected from the mouse ear canal after 5 days of infection (lower panel). BODIPY 493/503 staining assays were performed (**b**, **c**). BF brightfield. Scale bar, 10  μm.

Several studies reported the isolation of *C. auris* strains from the human ear canal^3,32–34^, and it is known that *C. auris* has a strong ability to colonize the human skin^35^. To determine whether there is a link between the ability to colonize skin and lipid droplet formation in *C. auris*, we performed in vivo assays using a mouse outer ear colonization model. *C. auris* cells were inoculated into the ear canals of mice (Fig. 2a). After 5 days of infection, *C. auris* cells were recovered from the ear canals and subjected to BODIPY 493/503 staining. We found that a large proportion of *C. auris* cells formed gLDs (approximately 35% for haploid cells and 25% for diploid cells; if the diameter of LDs was ≥ half of that of the cells, the LDs were defined as gLD) during the period of mouse ear colonization. The size of gLDs formed in vivo in the ear canals of mice was much larger than that of gLDs formed in vitro on the medical supplies tested (Fig. 2b and Supplementary Fig. 4), suggesting that the ear canal could be a natural niche that facilitates gLD formation in *C. auris*.

### *C. auris* cells with gLDs exhibit enhanced survivability under stressful in vitro conditions

**Fig. 3:**
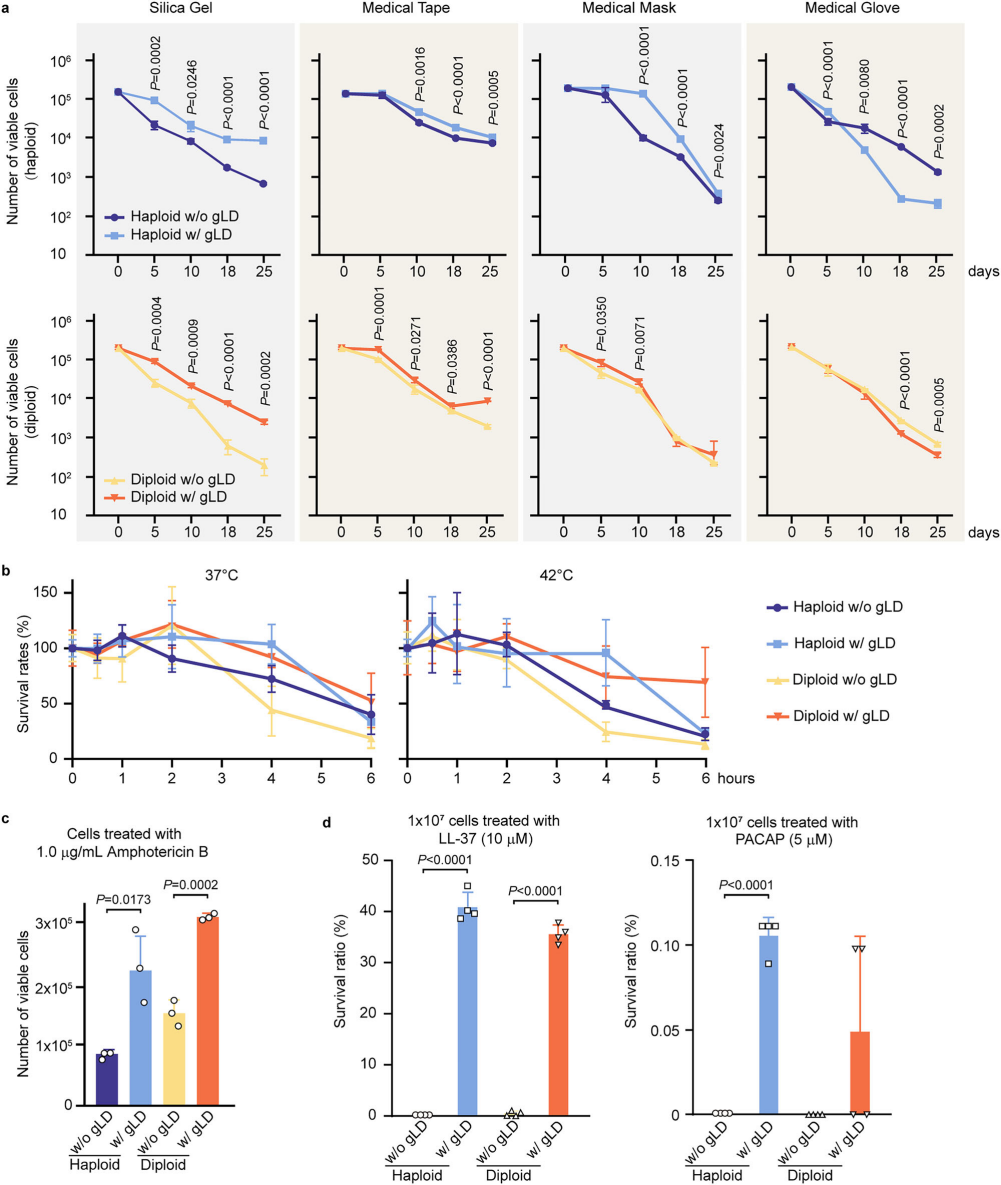
Comparative analysis of survivability of *C. auris* cells with (w/) or without (w/o) gLDs under stressful conditions. Haploid and diploid cells of strain AR0386 were examined under temperature, antifungal drug, and antimicrobial peptide stressors. *C. auris* cells w/ or w/o gLDs were collected from the cultures incubated on Kleyn or mKleyn media (w/o NaAc and w/ NaH_2_PO_4_) at 30 °C for 7 days, respectively. Significant differences were determined using two-tailed, unpaired Student’s *t* tests. **a** Survival curves of *C. auris* cells on the surfaces of silica gel, medical tape (cotton), masks (polypropylene), and gloves (butyronitrile). *C. auris* cells (5 × 10^6^ cells in 3 μL ddH_2_O) were spotted onto the surfaces of the medical supplies. CFU assays were performed on days 5, 10, 18, and 25 (note, a logarithmic scale is used for the y-axis). Three replicates were performed. **b** Survival curves of haploid and diploid cells in ddH_2_O at 37 °C and 42 °C. Approximately 2 ×10^5^ cells were suspended in 1 mL ddH_2_O in 1.5 mL Eppendorf tubes for each test. The tubes were then incubated in water bath at 37 °C and 42 °C for 6 h and the CFU assays were performed each hour. Three replicates were performed. **c** CFU assays of *C. auris* cells treated with amphotericin B (AmB). Approximately 1000 cells w/ gLDs or w/o gLDs were resuspended in 100 μL RPMI 1640 medium and mixed with 100 μL RPMI 1640 medium containing different concentrations of AmB in a 96-well plate and incubated at 30 °C for 24 h. CFU assays were then performed on YPD medium. **d** Survival rates of haploid and diploid cells treated with antimicrobial peptides LL-37 or PACAP. Approximately 1 × 10^7^ *C. auris* cells w/ gLD or w/o gLD cells were suspended in 1 mM potassium phosphate buffer (PPB) and treated with 10 μM LL-37 or 5 μM PACAP at 37 °C for 1 h. The cells were diluted and plated onto YPD medium for CFU analysis. Each dot represents a biological replicate; error bars represent standard deviations.

Since *C. auris* cells were able to form gLDs on medical supplies and in the ear canals of mice, we next examined whether gLDs can enhance fungal survivability under stressful in vitro conditions. *C. auris* cells with and without gLDs were collected from the Kleyn and mKleyn media, respectively, and were inoculated onto the surfaces of silica gel, medical tape, masks, and gloves. *C. auris* cells were recovered from these medical supplies and plated onto YPD medium for CFU assays after 5, 10, 18, and 25 days of incubation at 30 °C. Both haploid and diploid cells of *C. auris* containing gLDs exhibited an overall increased survivability on the surfaces of the medical supplies tested compared to those without gLDs (Fig. 3a). One exception was that haploid cells containing gLDs exhibited slightly reduced survivability on the surfaces of medical gloves (butyronitrile) after 10 days of incubation. Propidium iodide (PI, for dead cells) and BODIPY 493/503 staining assays further verified that the formation of gLDs clearly increased the survival of *C. auris* (Supplementary Fig. 6).

Two other notable characteristics of *C. auris* are thermotolerance and antifungal resistance^4,36^. We next tested the survivability of *C. auris* cells with and without gLDs when incubated in ddH_2_O at 37 °C and 42 °C. Both haploid and diploid cells containing gLDs exhibited higher survival rates than those lacking gLDs (Fig. 3b). Further investigation demonstrated that *C. auris* cells with gLDs were more tolerant to the antifungal drug amphotericin B (AmB, Fig. 3c) and the human antimicrobial peptides LL-37 and PACAP (Fig. 3d). Antimicrobial peptides LL-37 and PACAP have been shown to display strong antifungal activities against pathogenic fungi such as *C. albicans* and *C. auris*^22^. LL-37 and PACAP are produced mainly by keratinocytes or by other innate immune cells on human skin in response to inflammatory stimuli, which can disrupt cytoplasmic membranes^37^, and inhibit DNA, RNA, or protein synthesis of the pathogen on the skin^38–40^. Altogether, our findings suggest that gLDs play an important role in enhancing the survival rate of *C. auris* cells under stressful in vitro conditions.

### *C. auris* cells containing gLDs exhibit increased tissue colonization in both systemic and skin infection mouse models

**Fig. 4:**
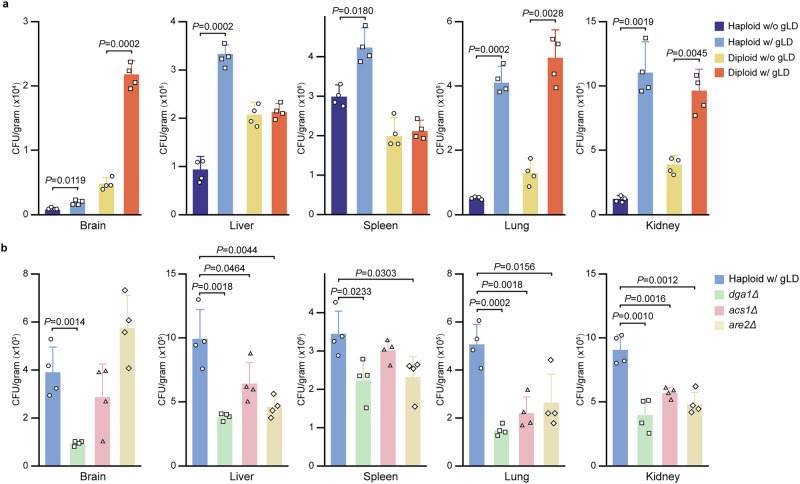
Fungal burdens of *C. auris* cells with (w/) or without (w/o) gLDs in a mouse model of systemic infection. **a** Haploid and diploid cells of strain AR0386 w/ or w/o gLDs. *C. auris* cells w/ or w/o gLDs were collected from the cultures incubated on Kleyn or mKleyn media (w/o NaAc and w/ NaH_2_PO_4_) at 30 °C for 7 days, respectively. **b** Haploid cells of the WT (AR0386) and *dga1*∆, *acs1*∆, and *are2*∆ mutant strains. *C. auris* cells with gLDs were collected from the cultures incubated on Kleyn media at 30 °C for 7 days. *C. auris* cells (2 × 10^7^) were injected into each mouse via the tail vein. Four mice were used for each strain sample. Fungal burdens of the brain, liver, spleen, lung, and kidney were analyzed after 24 h of infection. Significant differences were determined using two-tailed, unpaired Student’s *t* tests. Dots represent the CFUs; error bars represent standard deviations.

We next evaluated the virulence and colonization abilities of *C. auris* cells with and without gLDs. *C. auris* haploid and diploid cells were injected into mice via the tail vein. Fungal burden assays were performed after 24 h of infection. The fungal burdens of *C. auris* haploid cells with gLDs were significantly higher than those without gLDs in the five tissues examined (brain, liver, spleen, lung, and kidney, Fig. 4a). For *C. auris* diploid cells, a similar pattern in fungal burdens between cells with and without gLDs was observed in the lung, kidney, and brain, while no significant differences in fungal burdens were observed in the spleen and liver. Interestingly, compared to haploid cells, *C. auris* diploid cells with gLDs had a notable increased fungal burden in the brain.

*C. auris* is considered to be a skin colonizer, which can be easily transmitted in hospital environments, leading to outbreaks^41,42^. We next tested whether the development of gLDs could impact its capacity to colonize skin. *C. auris* haploid and diploid cells with and without gLDs were inoculated onto the dorsal back skin of newborn mice. CFU assays demonstrated that both haploid and diploid *C. auris* cells with gLDs exhibited a stronger skin colonization ability than those without gLDs (Supplementary Fig. 7).

### Lipidomic analysis of *C. auris* cells with and without gLDs

**Fig. 5:**
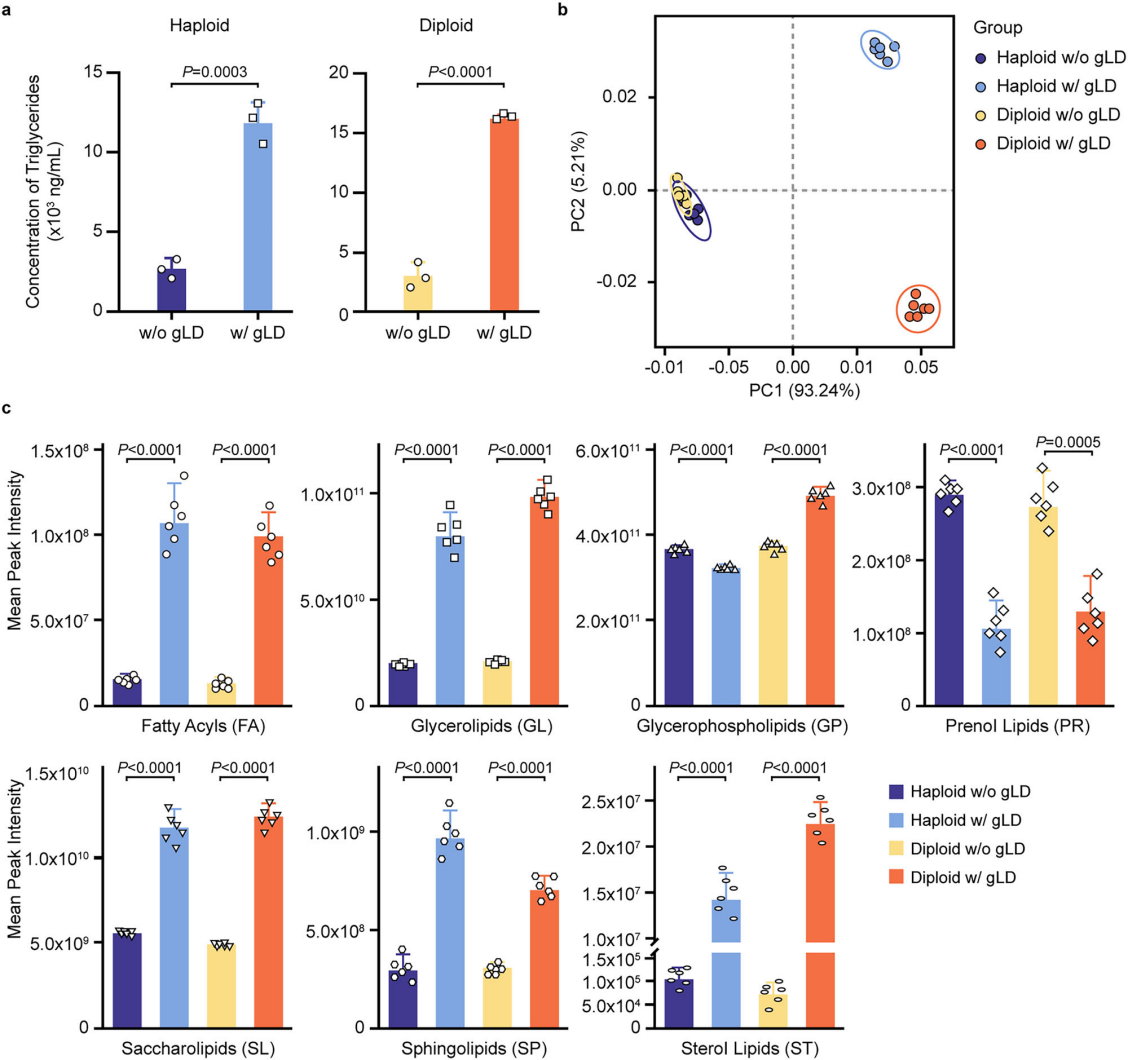
Lipid components of *C. auris* cells with (w/) or without (w/o) gLDs. Haploid and diploid cells w/ or w/o gLDs were examined. Significant differences were determined using two-tailed, unpaired Student’s *t *tests. **a** Level of triglyceride (TG). Approximately 1 × 10^7^ cells were collected, washed with PBS, and subject to sonication in 100 μL ice-cold TG assay buffer for cell wall disruption. The level of TGs was examined with the high sensitivity Triglyceride (TG) Assay Kit (Cat. Num. MAK264, Sigma-Aldrich). **b** Principal component analysis (PCA) score graphs of the four samples (haploid cells w/ or w/o gLDs, and diploid cells w/ or w/o gLDs). PC1 and PC2 represent the first and second principal component, respectively. The percentage represents the interpretation rate of the principal component in the dataset, whereas the ellipses represent a 95% confidence interval. Each dot represents a biological replicate of a certain sample. The six biological replicates of the samples w/ or w/o gLDs were separately plotted. **c** Relative levels of seven major lipid types in haploid and diploid cells w/ or w/o gLDs. The y-axis represents the mean peak intensity of lipids in each group. Dots represent the parallels; error bars represent standard deviations (**a**, **c**).

Previous studies indicated that triacylglycerols (TAG) and sterol esters are major components of lipid droplets^43^. As expected, we found that the level of TAGs in *C. auris* haploid and diploid cells with gLDs was significantly higher than that of cells without gLDs **(**Fig. 5a**)**. We next performed lipidomic analysis to explore the detailed lipid content differences between cells with and without gLDs. Principal component analysis (PCA) demonstrated that there were notable differences among haploid and diploid *C. auris* cells with and without gLDs (Fig. 5b). Analysis of the major categories of lipids indicated that the levels of fatty acyls (FA), glycerolipids (GL), sphingolipids (SP), sterol lipids (ST), and saccharolipids (SL) in *C. auris* cells with gLDs were significantly higher than in cells without gLDs (Fig. 5c). In contrast, the level of prenol lipids (PR) was comparatively higher in *C. auris* cells without gLDs. A detailed analysis of subclasses of lipid categories is shown in Supplementary Fig. 8 and Supplementary Data 1. In general, the levels of fatty acids, TAGs, steroid, phosphatidyl serines, and glycosphingolipids in both *C. auris* haploid and diploid cells with gLDs were higher than in those without gLDs (Supplementary Fig. 8a). In contrast, the levels of polyunsaturated fatty acids containing phospholipids (PUFA-PL), such as PUFA-phosphatidyl choline and PUFA-phosphatidyl ethanolamine were much lower in cells with gLDs than in cells without gLDs (Supplementary Fig. 8b). These results demonstrate that *C. auris* cells with and without gLDs have notable differences in both the components and levels of lipids.

### Transcriptome analysis of *C. auris* cells with and without gLDs

**Fig. 6:**
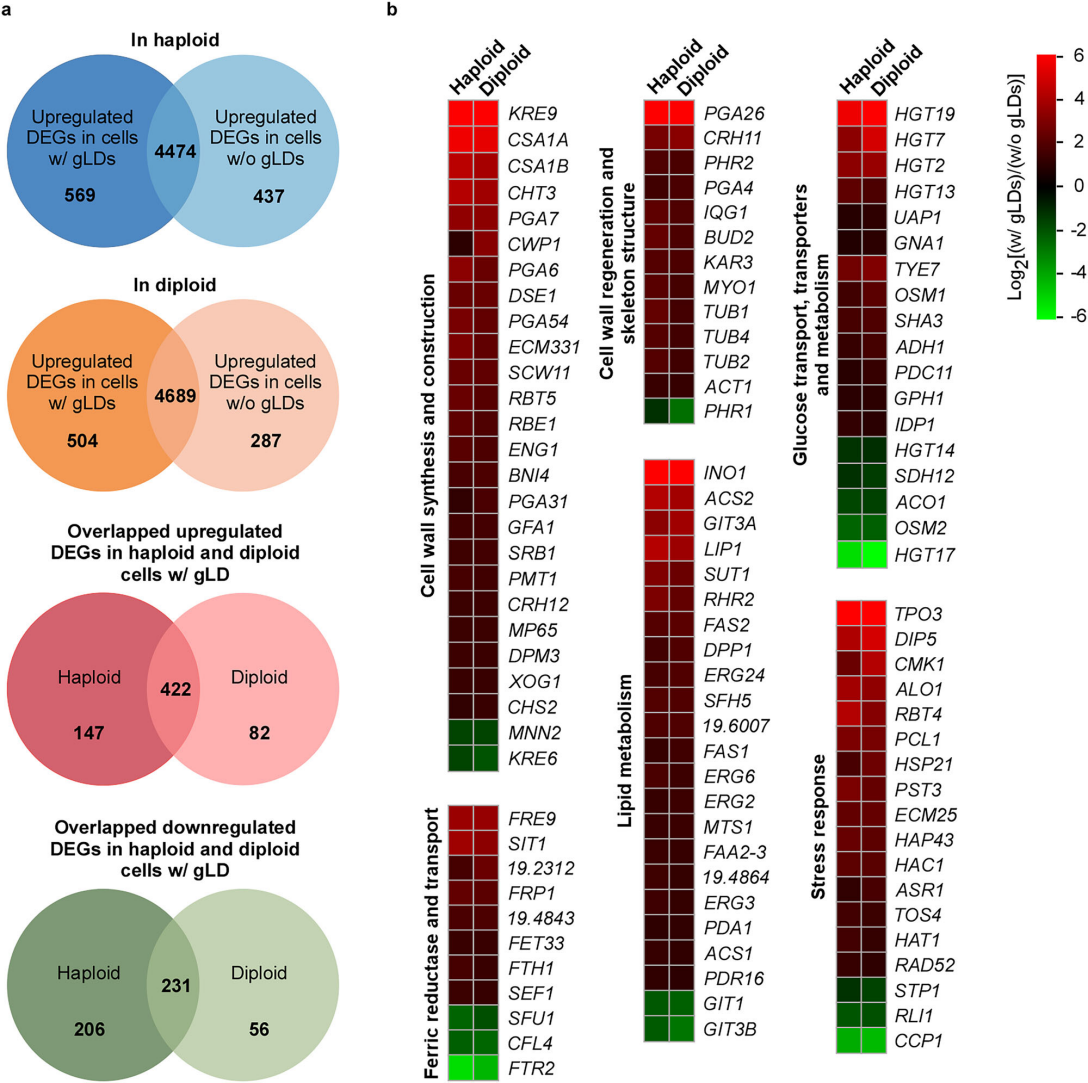
Differentially expressed genes (DEGs) between *C. auris* cells with (w/) or without (w/o) gLDs. Haploid and diploid cells w/ or w/o gLDs were cultured on Kleyn or mKleyn media, respectively. The cellular morphologies were examined to verify the formation of gLDs. **a** DEGs in haploid and diploid *C. auris* cells and common DEGs between haploid and diploid cells. **b** Representative DEGs involved in different biological processes. A twofold difference cut-off was used for DEGs.

To further understand the unique characteristics of *C. auris* cells with gLDs, we performed genome-wide transcriptional profiling by RNA sequencing (RNA-seq) on haploid and diploid *C. auris* cells of strain AR0386. *C. auris* cells were plated onto Kleyn medium and incubated at 30 °C for 4 days for the development of gLDs. Cells grown on mKleyn medium served as the control cells without gLDs. In total, we identified 1,006 and 791 differentially expressed genes between cells with and without gLDs in haploid and diploid strains, respectively (using a twofold difference cut-off, Fig. 6a). Among them, 653 common differentially expressed genes were identified in both the haploid and diploid comparative groups, indicating high consistency between different ploidy types. The differentially expressed genes are involved in the regulation of a number of biological processes such as cell wall synthesis and integrity, lipid and glucose metabolism, iron transportation, and stress responses (Fig. 6b and Supplementary Data 2). As expected, genes associated with lipid synthesis, including the genes of the acyl-CoA and TAG synthesis pathways, were significantly upregulated in *C. auris* cells with gLDs. Acyl-CoA synthetases are located in lipid droplets and are required for the formation of neutral lipids by providing fatty acyl-CoA substrates^43,44^. Putative acyl-CoA synthetase-encoding genes *ACS1, ACS2*, and *FAA2-3* and pyruvate dehydrogenase-encoding gene *PDA1* were upregulated in *C. auris* cells with gLDs. Also, expression of a number of genes of the TAG synthesis pathway (e.g., *FAS1, FAS2, RHR2, DPP1, orf19.6007*, and *orf19.4864*) was enriched in *C. auris* cells with gLDs. The increased expression levels of glucose transporter-associated genes could also contribute to de novo lipid or LD synthesis as observed in tumors or mammalian cells^45,46^. Moreover, we found that the expression of a number of genes associated with ferric reductases and iron transport (e.g., *SIT1*, *FRE9*, and *FRP1*) was increased in cells with gLDs. Of note, a similar increased expression pattern of iron metabolism-associated genes was observed during the development of LDs in *C. albicans*^47,48^, suggesting that the formation of LDs could be a conserved regulatory mechanism between the two species.

The ergosterol biosynthesis and sphingolipid synthesis pathways are generally important fungal pathways involved in antifungal resistance and environmental stress adaptation^49–51^. We found that a subset of genes involved in these two pathways (such as *ERG24, ERG6, ERG3, SUT1*, and *MTS1*) were upregulated in *C. auris* cells with gLDs. These findings are consistent with our results showing that *C. auris* cells containing gLDs display increased abilities to survive harsh environmental conditions.

Consistent with our observations that the cell walls of *C. auris* cells containing gLDs are thickened and remodeled/rebuilt (Fig. 1e), our RNA-seq analyses demonstrated that highly expressed cell wall-associated genes in *C. auris* cells with gLDs included *KRE9* (encoding a glycoprotein required for cell wall beta-glucan assembly), *ENG1* (encoding an endo-1,3-beta-glucanase), *XOG1* (encoding an exo-1,3-beta-glucanase), and chitin synthase-encoding genes *CHS2* and *CHS3*. Moreover, multiple genes involved in the regulation of glucose transportation (e.g., *HGT19, HGT7, HGT2*, and *HGT13*) and iron metabolism (e.g., *SIT1, FRE9, FRP1*, and *orf19.4843*) were upregulated in *C. auris* cells with gLDs.

We also found that a subset of genes was downregulated in *C. auris* cells with gLDs. These genes are involved in the processes of amino acid synthesis (e.g., *LEU1* and *LEU4, LYS4* and *LYS144*, and *ALT1*, for leucine, lysine, and alanine biosynthesis, respectively), amino acid transport (e.g., *GAP2, GPT1*, and *PUT4*), and protein translation (*DED1, RSM22, orf19.7254*, and *orf19.4779*). It remains to be investigated whether this downregulation of amino acid metabolism and protein translation is a protective strategy for *C. auris*. A detailed description of differentially expressed genes is presented in Fig. 6 and Supplementary Data 2. Overall, RNA-seq results indicate that multiple signaling and/or biosynthesis pathways could be involved in the regulation of lipid droplet formation in *C. auris*.

### Genetic regulation of gLD formation in *C. auris*

**Fig. 7:**
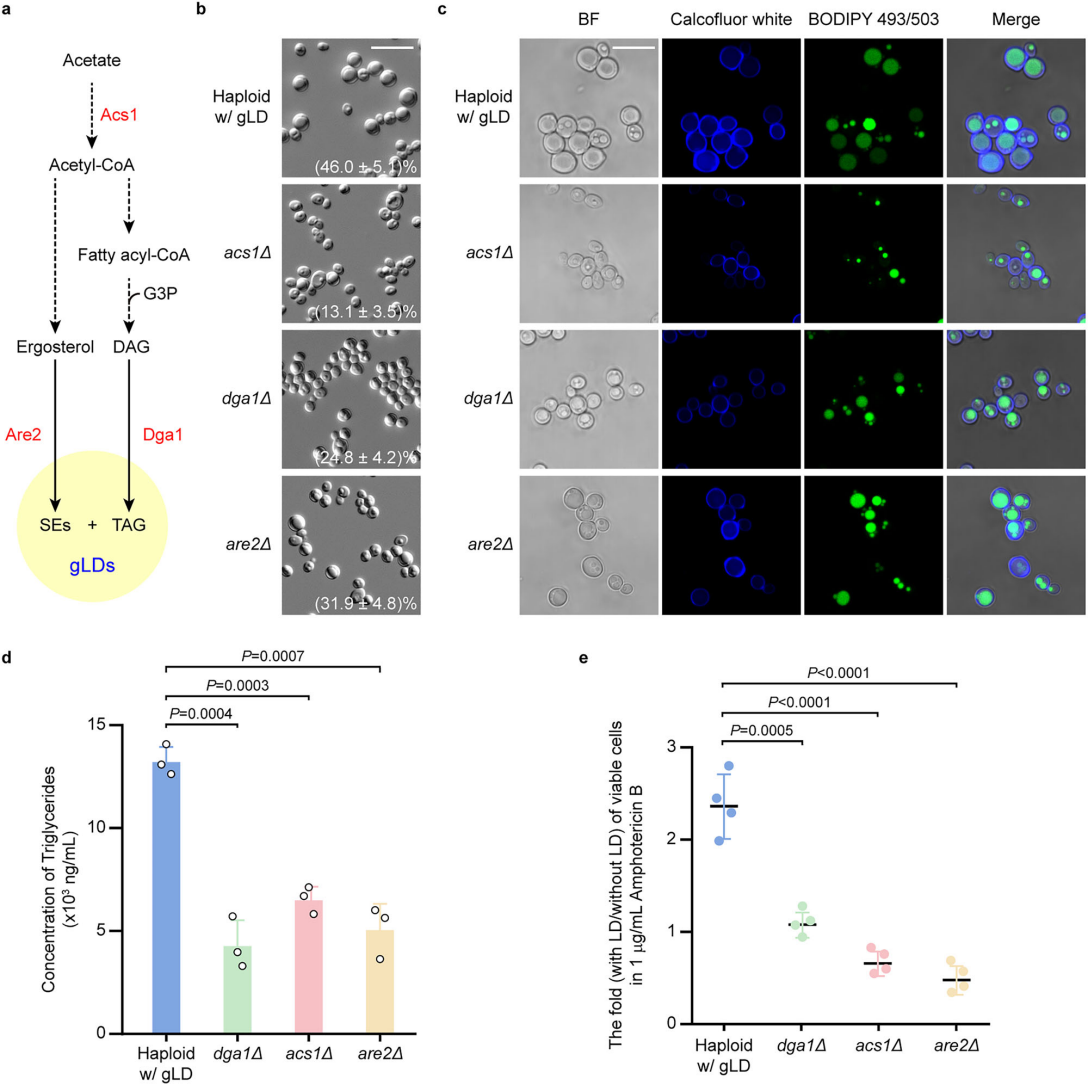
Acs1, Dga1, and Are2 regulate the formation of gLDs in *C. auris.* **a** Diagram of the TAG and SE synthesis pathway. **b**, **c** Cellular morphologies of the WT (AR0386), *acs1*∆, *dga1*∆, *and are2*∆ mutant strains*. C. auris* cells were initially grown on YPD medium at 30 °C for 2 days and then replated onto Kleyn medium and incubated at 30 °C for 7 days. The percentage of cells with gLDs is shown in each image (**b**). BF, brightfield. Scale bar, 10 μm. **b** DIC images; c, Calcofluor white and BODIPY 493/503 staining assays. **d** Comparative analysis of the level of triglyceride. **e** Comparative analysis of survivability of *C. auris* cells treated with amphotericin B. CFU assays were performed. Significant differences were determined using two-tailed, unpaired Student’s *t* tests. Error bars represent standard deviations (**d**, **e**).

The synthesis of TAGs and SEs is acetyl-CoA-dependent and requires the acetyl-CoA synthetase Acs1 (Fig. 7a). The diacylglycerol acyltransferase Dga1 and acyl-CoA:sterol acyltransferase Are2 are key enzymes for the production of TAGs and SEs, respectively^52^. To explore the molecular bases for the development of gLDs in *C. auris*, we deleted a selection of genes associated with the synthesis of TAGs and SEs, representing the two major components of lipid droplets^53,54^. We found that deletion of *ACS1, DGA1*, and *ARE2* clearly attenuated LD formation (Fig. 7b,c,andSupplementary Fig. 9). Deletion of *ACS1* has the strongest impact on LD formation, where the *acs1* mutant strain exhibited the weakest ability to form lipid droplets. Consistently, the cell wall chitin levels of these mutant strains were also remarkably decreased as demonstrated by calcofluor white staining assays (Fig. 7c). Compared to the wildtype control, the levels of TAGs in the *dga1*, *acs1*, and *are2* mutant strains were also significantly lower (Fig. 7d).

We next examined the survivability of the *dga1*, *acs1*, and *are2* mutant strains under stressful in vitro conditions (Fig. 7e and Supplementary Fig. 10). When treated with amphotericin B (Fig. 7e) or when inoculated on the surfaces of silica gel, medical tape, masks, or gloves (Supplementary Fig. 10), the *dga1*, *acs1*, and *are2* mutant strains exhibited significantly reduced survivability relative to the wildtype control strain. Consistently, the mutant strains generally demonstrated decreased abilities to colonize tissues in a mouse systemic model based on fungal burden assays (Fig. 4b). Interestingly, however, the *are2* mutant strain exhibited an increased fungal burden in the brain. The underlying reason for this tissue-specific increased fungal burden remains to be investigated. Altogether, our results indicated that the TAG and SE synthesis pathways are not only important for the development of gLDs but also for the survival of *C. auris* under stressful conditions both in vivo and in vitro.

Since acetyl-CoA and mitochondrial metabolism play critical roles in central carbon metabolism in fungi^55,56^, we next explored the effects of inactivation of mitochondrial metabolism on the development of lipid droplets in *C. auris*. The mitochondrial protein Mcu1 (Multiple Carbon source Utilizer 1) is required for mitochondrial metabolism and virulence in *C. albicans* and *C. auris*^56,57^. We found that deletion of *MCU1* nearly completely blocked the formation of gLDs in *C. auris* (Supplementary Figs. 9, 11). Consistently, inactivation of *CIT1* (encoding a rate-limiting enzyme of the TCA cycle, citrate synthase) or *SDH2* (encoding a succinate dehydrogenase) had similar effects on the formation of gLDs. These findings indicate that mitochondrial metabolism is required for gLD formation in *C. auris*.

## Discussion

It has been suggested that the striking capacity of *C. auris* for skin colonization and environmental persistence could be factors contributing to the rapid spread and outbreaks of *C. auris* in clinical settings^6,41,58^. Natural *C. auris* strains often exhibit high thermotolerance and salinity tolerance^59^. Casadevall et al. ^13^ proposed that the emergence of *C. auris* could be linked to climate change and its original ecological niche could be coastal wetlands^13^. Adaptation to natural niches could shape the unique biological features of *C. auris;* however, it remains unclear how this pathogen adapts to its host and stressful environments as well as the biological and genetic bases for its enhanced ability to survive in vivo and in vitro stresses. In this study, we report that *C. auris* forms giant lipid droplets (gLDs) to increase its ability to survive under stressful conditions. *C. auris* cells containing gLDs undergo cell wall remodeling and fundamental global transcriptional changes and demonstrate increased abilities to colonize the host and to survive in the environment. Inactivation of the TAG and SE synthesis pathways or genes essential for mitochondrial metabolism attenuated or blocked the development of gLDs in *C. auris*. Consistently, the associated mutant strains exhibited decreased survivability under stressful conditions. Our findings uncover a new survival strategy for *C. auris* under harsh environments.

In eukaryotic cells, the primary roles of lipid droplets are for fat storage and energy homeostasis^60,61^. Recent research has indicated that LDs play critical roles in cellular stress response through buffering the levels of toxic lipid species and maintaining redox homeostasis^61–63^.

*C. auris* cells often encounter harsh stressors during both host infection and environmental colonization, such as nutrient deprivation, oxidative stress, and antimicrobial agents^64,65^. We found that in response to environmental stressors, *C. auris* cells can form gLDs and undergo cell wall remodeling (Fig. 1). Among the stressors, nutrient limitation could be a major signal for the induction of gLD formation. We found that, other than the nutrient-poor sporulation medium (Kleyn medium), *C. auris* cells formed gLDs under extreme nutrient deprivation conditions such as on the surfaces of water agar (medium containing only 4% agar with water and no added nutritional components) as well as on medical supplies (Supplementary Figs. 2 and 4). In turn, gLDs protect *C. auris* cells and enhance fungal cell viability when the cells are exposed to stressful environments. For example, *C. auris* cells with gLDs survived much better both on the surface of medical supplies and on the skin of mice than cells without gLDs (Fig. 3a and Supplementary Fig. 7). Moreover, *C. auris* cells with gLDs also showed an increased tolerance to the human antimicrobial peptides LL-37 and PACAP and a significantly enhanced fungal burden in a mouse systemic infection model (Figs. 3d, 4a). LL-37 and PACAP are generally involved in skin immunity; in addition, LL-37 can stimulate the production of cytokines and activate glial cells in the brain, which is important for innate cerebral immunity; and PACAP has been shown to have a neuroprotective effect on the brain^66,67^. These findings indicate that the formation of gLDs in *C. auris* is not only associated with environmental persistence but is also associated with virulence and tolerance of antimicrobial peptides during host infection. These unique biological characteristics of *C. auris* could contribute to its rapid transmission and potential to cause outbreaks in hospital settings.

To explore the potential underlying protective mechanisms of gLDs, we performed lipidomic and transcriptomic analyses on gLDs (Figs. 5, 6). In eukaryotic cells, LDs store neutral lipids that not only serve as reservoirs of neutral lipids but also prevent cellular lipotoxicity by the sequestration of excess lipids^68^. In certain mammalian cell types, reactive oxygen species (ROS) can induce LD formation^69^. In turn, LDs play a critical role in detoxifying ROS and resolving intracellular oxidative stress^70^. RNA-Seq analysis demonstrated that the expression of mitochondrial metabolism or oxidative metabolism-associated genes were enriched in *C. auris* cells containing gLDs (Fig. 6 and Supplementary Data 2), indicating a potential link between oxidative stress and the formation of gLDs. Consistent with this hypothesis, we found that inactivation of genes required for the central node of LD formation (*ACS1, DGA1*, and *ARE2*), or genes essential for mitochondrial metabolism, attenuated or blocked LD formation in *C. auris* (Fig. 7, Supplementary Figs. 9, 11) and led to reduced survivability under stressful conditions both in vivo and in vitro (Fig. 4 and Supplementary Fig. 10). It remains to be investigated how environmental stresses signal gLD formation through the lipid synthesis and mitochondrial metabolism pathways.

The levels of several lipid categories or precursors, such as FA, GL, SP, ST, and SL, were significantly increased in *C. auris* cells containing gLDs (Fig. 5). Of them, GP and SP are crucial to many biological processes including virulence, cell wall integrity and mitochondrial dysfunction in pathogenic fungi^50,51^. In addition, saturated, monounsaturated and polyunsaturated FAS (PUFAs) have been indicated to be involved in membrane remodeling and composition in cells experiencing stress^71–73^. We also observed that the cell wall underwent remarkable remodeling in *C. auris* cells containing gLDs (Fig. 1 and Supplementary Fig. 3). Consistently, many genes involved in cell wall integrity and regeneration were highly upregulated (Fig. 6b). In addition, several iron metabolism-associated genes were also upregulated in *C. auris* cells containing gLDs. Previous studies found that iron limitation could lead to beta-glucan masking and cell wall remodeling in *C. albicans*^74^, indicating potential links between LD formation, cell wall integrity, and iron metabolism. In addition, the thickened cell wall and increased levels of neutral lipids in *C. auris* cells containing gLDs are somewhat structurally reminiscent of *C. albicans* chlamydospores^27^. Interestingly, Lopes-Bezerra et al.^75^ reported a similar bi-layered cell wall structure of *Sporothrix schenckii*, a pathogenic fungus causing sporotrichosis^75^. The outer layer of the *S. schenckii* cell wall was also shed under certain culture conditions. Here we observed that the outer layer of the *C. auris* cell wall detached from the cells under harsh in vitro and in vivo conditions (Supplementary Fig. 12). This process occurred concurrently with the formation of gLDs and a new thickened cell wall. Overall, the changes in lipid components and cell wall structures found in *C. auris* cells containing gLDs could directly affect cellular physiology and thus enhance *C. auris*’ tolerance to environmental stresses.

In summary, microbial pathogens often use multiple strategies to survive under stressful or harsh conditions. Here we report that *C. auris* cells form gLDs and undergo cell wall remodeling to adapt to in vivo and in vitro harsh environments. *C. auris* cells containing gLDs have a thickened cell wall and resemble fungal spores in many biological aspects, including transcriptional expression profiles, and in terms of tolerance to environmental stresses and antifungal drugs and peptides. These gLD containing cells could, therefore, represent a new cell type of *C. auris* with unique biological characteristics. Together with its ability to form biofilms and other notable virulence traits, the ability to form gLDs could be another important biological feature that contributes to *C. auris* being a “superbug” fungal pathogen. Despite the many studies that demonstrate that LDs are involved in the regulation of stress adaptations in eukaryotic organisms, it remains to be investigated exactly how gLDs regulate the increased survivability and tolerance to stressors in *C. auris*. What is the link between the formation of gLDs and cell wall remodeling? How does the thickened cell wall coordinate with gLDs to cope with stressful environments? Altogether, our findings reveal a new biological feature of this emerging fungal pathogen. Further mechanistic investigations into *C. auris* gLDs will benefit the development of novel strategies to prevent *C. auris* transmission and/or treat *C. auris* infection in the future.

## Materials and methods

### Culture conditions and morphological analysis assays

YPD medium (20 g/L peptone, 10 g/L yeast extract, and 20 g/L glucose) was used for routine growth of *C. auris*. Kleyn medium (2.5 g/L peptone, 0.62 g/L glucose, 0.62 g/L NaCl, and 5 g/L NaAc) was used for the development of lipid droplets in *C. auris*. Modified Kleyn medium (mKleyn, Kleyn medium deprived of NaAc and with 0.6 g/L NaH_2_PO_4_) was used as a reference medium to repress the formation of lipid droplets. For solid medium, 20 g/L agar (BD Bacto™ Agar, BD Biosciences) was added. For the induction of lipid droplet formation, *C. auris* cells were initially incubated on YPD media at 30 °C for 2 days, then approximately 5 × 10^6^ cells in 10 μL ddH_2_O were spotted onto Kleyn medium and cultured at 30 °C for 7 days. *C. auris* cells grown on mKleyn medium served as controls.

### Strains used and construction of deletion mutant strains

Haploid and diploid strains of the four major clades (BJCA001, I; CBS12373, II; RICU2, III; and AR0386, IV) were used in this study. The *acs1*∆, *dga1*∆, and *are2*∆ mutant strains were constructed in *C. auris* strain AR0386, and *cit1*∆, and *sdh2*∆ mutant strains were generated in strain BJCA001^16^. The fusion PCR product recombination strategy^76^ was used for gene deletion with a *caSAT1* positive selection marker, a *Candida*-adapted *SAT1*^77^. Primer pairs (caSAT1 FWD/caSAT1 REV) were used to amplify the *caSAT1* cassettes from plasmid pSFS2A by PCR. For example, to delete the gene *ACS1*, the fusion PCR products of the *caSAT1* cassette flanked by the 5’- and 3’- fragments of *ACS1* were transformed into strain AR0386. For positive selection of transformants, 500 µg/mL nourseothricin (clonNAT) was added to YPD solid medium. Correct transformants were verified by PCR assays.

The *mcu1*Δ mutant strain was constructed in strain BJCA001 using plasmid pSFS2A^77^. The upstream and downstream flanking sequences of *MCU1* were amplified from genomic DNA of strain BJCA001 and subcloned into plasmid pSFS2A at the *Kpn*I/*Sal*I and *Not*I/*Sac*II sites, respectively, generating the deletion plasmid pSFS2A-auMCU1. Linearized plasmid pSFS2A-auMCU1 (with *Kpn*I and *Sac*II) was then transformed into strain BJCA001, generating the *mcu1*∆ mutant strain. For positive selection of transformants, 500 μg/mL clonNAT was added into YPD solid medium. Correct transformants were verified by PCR assays. Primers used for mutant strain construction are listed in Supplementary Data 3. Wild type and mutant strains are listed in Supplementary Data 4.

For *C. auris* transformations, cells were initially cultured in 2 mL of YPD liquid medium with shaking for overnight growth. Then, 400 μL of the culture was inoculated to 20 mL of fresh YPD liquid medium and grown to mid-logarithmic phase (OD_600_ = 1.8 to 2.0). Fungal cells were collected for electroporation assays.

### Microscopy assays

Differential interference contrast (DIC) assays were used for regular cellular morphology analysis. For nuclear DNA staining, 4’,6-diamidino-2-phenylindole (DAPI, Sigma-Aldrich) was used at the final concentration of 2 μg/mL. Propidium iodide (PI, Sigma-Aldrich) staining assays were used to evaluate cell viability at the final concentration of 25 μg/mL. For cell wall chitin staining, Calcofluor white (Siama-Aldrich) was used at the final concentration of 10 μg/mL. BODIPY 493/503 (Invitrogen, 2 μg/mL) and Nile Red (APExBio, 10 μg/mL) were used for lipid droplet staining. Stained *C. auris* cells were then imaged using the laser scanning microscopes LSM710 (Zeiss, German).

For scanning electron microscopy (SEM) assays, *C. auris* diploid cells (AR0386) were first fixed with 2 × electron microscope fixative for 5 min and then transferred into 1 × electron microscope fixative for 2 h at 4 °C. The samples were dehydrated with gradually increasing concentrations of ethanol (50%, 75%, and 90%) for 10 min, respectively, and then with 100% ethanol for 10 min for three times. A de-alcohol assay was then performed with gradually increasing concentrations of tert-butanol (50%, 75%, and 90%) for 10 min, respectively, and with 100% tert-butanol for 10 min three times. The samples were dried and coated with gold, and imaged using the scanning electron microscope FlexSEM 1000 (Hitachi, Ltd., Japan).

For transmission electron microscopy (TEM) assays, *C. auris* diploid cells (AR0386) were suspended in the TEM fixative and fixed at 4 °C. TEM assays were performed by Wuhan Servicebio Technology CO., LTD. In brief, the treated samples were embedded into resin and the resin blocks were sliced as thin as 60–80 nm on the ultramicrotome (Leica UC7 with Daitome Ultra 45° diamond slicer) and fished out onto the 150 meshes cuprum grids with formvar film. Then, cuprum grids were stained in 2% uranium acetate and imaged with the Talos L120C TEM microscope (Thermo Fisher Scientific).

### Survival assays

*C. auris* cells were initially incubated on YPD solid medium at 30 °C for 2 days and then inoculated onto Kleyn or mKleyn medium (5 × 10^6^ cells in 10 μL ddH_2_O) and incubated at 30 °C for 7 days. For survival experiments on medical supplies, *C. auris* cells with gLDs (from Kleyn medium) or without gLDs (from mKleyn medium) were suspended in ddH_2_O and 5 × 10^6^ cells (3 μL) were spotted on different medical supplies. After 5, 10, 18, or 25 days of incubation at 30 °C, *C. auris* cells were recovered from the surfaces of the medical supplies with ddH_2_O. CFU assays were performed on YPD medium to evaluate the survival rate.

For antifungal treatment assays, approximately 1000 cells with or without gLDs were suspended in 100 μL RPMI 1640 medium (1.04% RPMI 1640, 3.45% MOPs, [wt/vol], NaOH used for adjustment to pH 7.0), then mixed with 100 μL RPMI 1640 medium containing different concentrations of amphotericin B in a well of 96-well plates and incubated at 30 °C for 24 h. Three biological repeats were performed for each strain. CFU assays were performed on YPD medium.

For survival assays under high-temperature conditions, approximately 200,000 cells with or without gLDs were suspended in 1 mL ddH_2_O, and incubated in water bath at 37 °C or 40 °C. After 30, 60, 120, 240, or 360 min of treatment, CFU assays were performed on YPD medium.

For human antimicrobial peptides (AMP) killing assays, approximately 1 × 10^7^ cells with or without gLDs were suspended in 1 mM potassium phosphate buffer (PPB) and treated with 10 μM LL-37 (APExBIO, Shanghai, China) or 5 μM PACAP 1-38 (ACMEC, Shanghai, China) at 37 °C for 1 h. Three biological repeats were performed. Then, treated cells were individually collected and plated onto YPD solid medium for CFU analysis to calculate the survival rate.

### Animal experiments

All animal experiments were performed in accordance with the guidelines approved by the Fudan University Animal Care and Use Committee (2021JS004).

All mice were purchased from Vital River (Beijing, China). Female BALB/c mice (6-weeks-old) were routinely raised in a P2 laboratory. All mice were acclimatized for two days under controlled conditions (21–23 °C, 50–70% relative humidity, 12 h light/dark cycle) with libitum access to food and water.

For mouse ear colonization assays, haploid and diploid cells of *C. auris* strain AR0386 were plated onto YPD solid medium at 30 °C for 2 days, collected, washed twice with 1 × PBS, and then suspended in 1 × PBS. Female BALB/c mice (6-weeks-old) were anesthetized via intraperitoneal injection of 100 μL 1% pentobarbital sodium (50 mg/kg body-weight dosing). Approximately 1 × 10^8 ^*C. auris* cells in 20 μL suspension were inoculated into the mouse ear canals. When the ear canal surface dried, the mouse auricle was wrapped and fixed with medical tape. The mice were humanely killed after 5 days of inoculation. *C. auris* cells were collected from the mouse auricle and re-suspended in 1 × PBS and stained with BODIPY 493/503. Three mice were used for each strain.

For fungal burdens assays, *C. auris* haploid and diploid cells with or without gLDs of strain AR0386 and haploid cells of the *acs1*∆, *dga1*∆, and *are2*∆ mutant strains were examined. Four female BALB/c mice (6-weeks-old) were used for each strain. *C. auris* cells (2 × 10^7^ cells in 250 µL 1 × PBS) were injected into the mouse lateral tail vein. After 24 h of infection, the mice were humanely killed by cervical dislocation. For CFU and fungal burden analyses, five organs (brain, liver, spleen, lung, and kidney) were collected, weighed, and ground using a tissue grinder (Scientz-48L, Ningbo Scientz Biotechnology Co.,LTD., China, with a frequency of 70 Hz for 120 s), and then plated onto YPD solid medium with 34 µg/mL chloramphenicol.

For mouse skin infection experiments, *C. auris* haploid and diploid cells with or without gLDs of strain AR0386 were examined, using newborn BALB/c mice (3–5-days-old). Approximately an area of 8 mm^2^ was marked on the dorsal back skin of the newborn mice for *C. auris* colonization. A suspension of 2 × 10^6^ fungal cells in 2 µL 1× PBS was spotted on the marked area after disinfection with 75% ethanol. The suspension was evenly spread on the marked area. A small sterilized glossy paper was affixed on the dried inoculated spot with First Aid waterproof tape. After 3 days of infection, the infected skin was excised and used for CFU assays.

### Triglyceride (TG) level test

*C. auris* cells were initially grown on YPD medium at 30 °C for 2 days and then spotted on Kleyn or mKleyn medium and incubated at 30 °C for 7 days. The level of TG was detected by the high sensitivity Triglyceride Assay Kit (Cat. Num. MAK264, Sigma-Aldrich). *C. auris* cells with or without gLDs (1 × 10^7^ cells) were suspended in 100 μL ice-cold TG assay buffer and subjected to ultrasound treatment (2 s ultrasound, and 2 s quiescence, totally of 10 min). Fluorescence intensity (l_ex_=535 nm/l_em_ = 587 nm) was measured by ELISA. Then the concentration of triglyceride was calculated (ng/μL, concentration=S_a_/S_v_, S_a_ = level of triglycerides in the sample from the standard curve, S_v_ = sample volumes added into the wall).

### Genome-wide transcriptional profiling by RNA sequencing (RNA-Seq)

C. *auris* cells grown on solid Kleyn (with gLDs) and mKleyn medium (without gLDs) at 30 °C for 7 days were used for total RNA extraction. RNA-Seq analysis was performed by Berry Genomics CO., (Beijing, China) with the Illumina NovaSeq platform. Approximately 3 GB reads were obtained by sequencing each library as described in our previous publications^78^. Low-quality (Phred score ≤10), ambiguous, and adaptor bases were removed from the raw reads using the FASTX-Toolkit v0.0.14 (http://hannonlab.cshl.edu/fastx_toolkit/index.html). The clean reads were aligned to the transcriptome of *C. auris* (from the NCBI database; accession number GCA_002759435.2)^79^ using the software HiSat2 v2.0.5 with default parameters. Transcriptional expression of different samples was estimated with StringTie v1.3.3b using default parameters^80^. GO functional enrichment analysis was carried out according to the online CGD GO Term Finder tool (http://www.candidagenome.org/cgi-bin/GO/goTermFinder). Differentially expressed genes (DEGs) were analyzed with the DESeq2 R package^81^. DEGs needed to satisfy three criteria: (a) an FPKM (fragments per kilobase per million) value for at least one sample of ≥ 20; (b) the absolute fold change of ≥ 2; and (c) false discovery rates (FDRs) of < 0.05.

### Lipidomic analysis

Haploid and diploid cells of *C. auris* strain AR0386 were initially grown on YPD plates at 30 °C for 2 days and then spotted on Kleyn or mKleyn medium and incubated at 30 °C for 7 days. *C. auris* cells with gLDs (on Kleyn) or without gLDs (on mKleyn) were weighed and used for lipidomic analysis (performed by BGI Genomics Co., Ltd.). Briefly, lipids were comprehensively detected by LC-MS/MS and LipidSearch (Thermo Fisher Scientific, USA) was used for identification of lipid types. Filling missing values, data normalization, and QC analysis were performed by metaX^82^. Variable importance in projection (VIP) was analyzed using the PLD-DA model and significantly different unsaturated lipids needed to satisfy three criteria: (a) VIP ≥ 1; (b) fold change ≥3; and (c) the unsaturated lipid of haploid and diploid remain consistent.

### Analysis of the relative composition of cell wall components

Cell wall components were determined according to the methods described by Ouyang et al.^83^. Briefly, haploid and diploid cells of strain AR0386 with (w/) or without (w/o) gLDs were examined. Cells w/ or w/o gLDs were collected from the cultures grown on Kleyn or mKleyn media, incubated at 30 °C for 7 days, respectively. Approximately 2 × 10^9^ cells were collected in 1 mL 50 mM NH_4_HCO_3_ (pH 8.0) containing 0.2 g glass beads (0.5 mm diameter). Cells were lysed using a Disruptor Genie (Scientific Industries) with the following settings: 5 min vibration, 3 min rest, repeated a total of 5 times. Fungal cell homogenates were then centrifuged and washed three times with ddH_2_O, and the cell wall suspensions were then incubated with 1 M KOH at 70 °C for 30 min to release the alkaline-soluble materials. Glycoprotein in the supernatant was precipitated with 2 volumes of ethanol, washed twice with 64% ethanol, and dissolved in distilled water. The alkaline-insoluble materials were washed with ddH_2_O several times and digested in 6 M HCl at 100 °C for 2 h to release monosaccharides from β-glucan and chitin. After digestion, HCl was evaporated and the residues were dissolved in 0.2 mL distilled water. The levels of β-glucans were estimated by measuring released glucose using the phenol/sulfuric acid method and the levels of chitins were determined by measuring N-acetylglucosamine. Three independent repeats of each *C. auris* cell sample were performed.

### Statistics and reproducibility

To assess whether the data met the assumptions of the statistical approach, we tested normality using the Shapiro-Wilk test and visualized data distribution with Q-Q plots. Homogeneity of variance was evaluated using Levene’s teat. Two-tailed, unpaired Student’s *t* test were used for Figs. 3, 4, 5, 7, S7, S8, and S10. GraphPad Prism v8 was used for all statistical analyses in this study.

The morphologies of *C. auris* colonies and cells (as observed by SEM, TEM, and DIC microscopy) presented in the figures were representative of at least three independent experiments. All source data underlying the graphs were listed in Supplementary Data 5.

### Reporting summary

Further information on research design is available in the Nature Portfolio Reporting Summary linked to this article.

## Supplementary information

Supplementary information-combined file

Description of Additional Supplementary Files

Dataset 1

Dataset 2

Dataset 3

Dataset 4

Dataset 5

Reporting summary

## Data Availability

The authors declare that the data supporting the findings of this study are available within the article and its Supplementary Information files. RNA-seq data have been deposited into the NCBI WGS bioproject PRJNA1158674. The SRA database and the accessible numbers are SRR30597570-SRR30597581. The source data of RNA-seq analysis are presented in Supplementary Data 2.
