# Supplementary material for: Candida auris cells form giant lipid droplets to survive in harsh environments
Source: Commun Biol. 2025 May 22;8:783. doi: 10.1038/s42003-025-08204-7 (PMC12098800; doi:10.1038/s42003-025-08204-7)
Supplement: Supplementary file 1 — Supplementary information-combined file [file 42003_2025_8204_MOESM1_ESM.pdf]

Supplementary information for

Candida auris cells form giant lipid droplets to survive in harsh environments

Qiushi Zheng[1#], Chao Li[1#], Tianren Hu[2,3#], Jian Bing[1], Clarissa J. Nobile[4,5], Haiqing Chu[2,3*], and Guanghua Huang[1,6*]

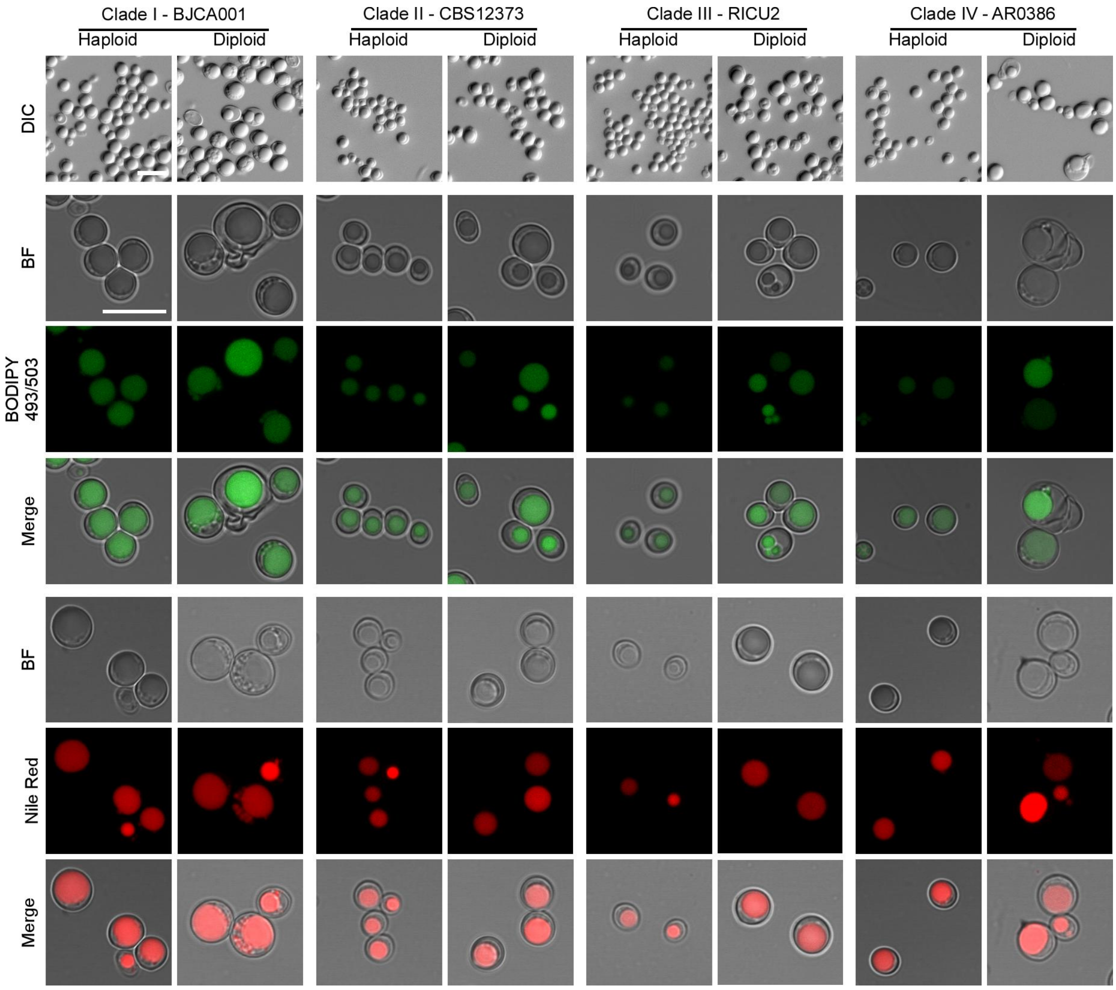

Supplementary Figure 1. Formation of gLDs in *C. auris* strains of the four major genetic clades.

BODIPY 493/503 and Nile red staining assays for lipid droplets were performed. Haploid and diploid cells of strains BJCA001 (Clade I), CBS12373 (Clade II), RICU2 (Clade III), and AR0386 (Clade IV) were initially grown on

YPD medium for 2 days at 30°C, and then spotted onto Kleyn medium (5 x 10^6 cells in 10 μL ddH_2O) and incubated at 30°C for 7 days. BODIPY493/503 and Nile Red staining assays for lipid droplets, respectively. BF, brightfield. Scale bar for DIC, 10 μm; scale bar for brightfield and stained cells, 10 μm.

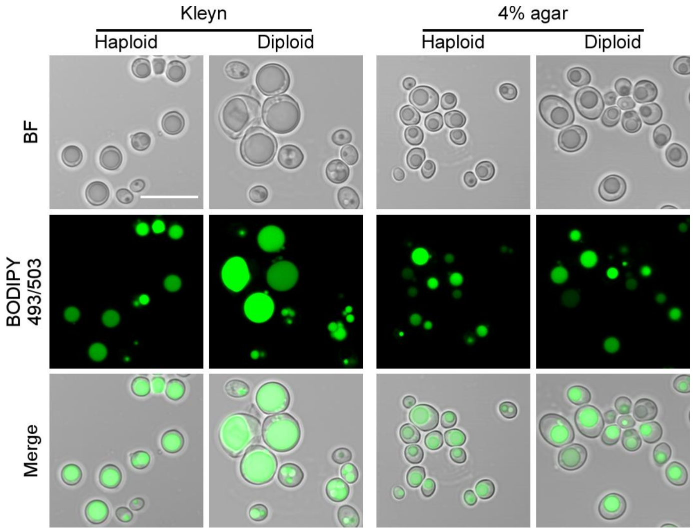

Supplementary Figure 2. Formation of gLDs in *C. auris* on Kleyn and water agar media.

C. auris haploid and diploid cells of strain AR0386 were initially grown on YPD medium for 2 days at 30°C, and then spotted onto Kleyn or water agar medium (5 x 10^6 cells in 10 μL ddH_2O) and incubated at 30°C for 7 days. The water agar medium contained only 4% agar (BD Bacto™ Agar, BD Biosciences) and no other nutritional components. *C. auris* cells were stained with BODIPY 493/503. BF, brightfield. Scale bar, 10 μm.

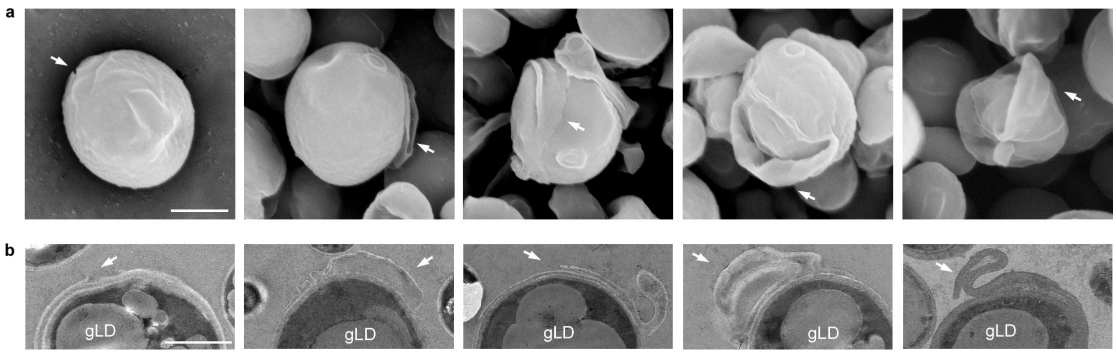

Supplementary Figure 3. SEM and TEM images of *C. auris* cells with gLDs.

C. auris diploid cells of strain AR0386 were initially grown on YPD medium for 2 days at 30°C, and then spotted onto Kleyn medium (5 x 10^6 cells in 10 µL ddH_2O) and incubated at 30°C for 7 days. (a) SEM images. (b) TEM images of the cell wall structure. Arrows indicate detached cell wall structures. Scale bar, 2 µm.

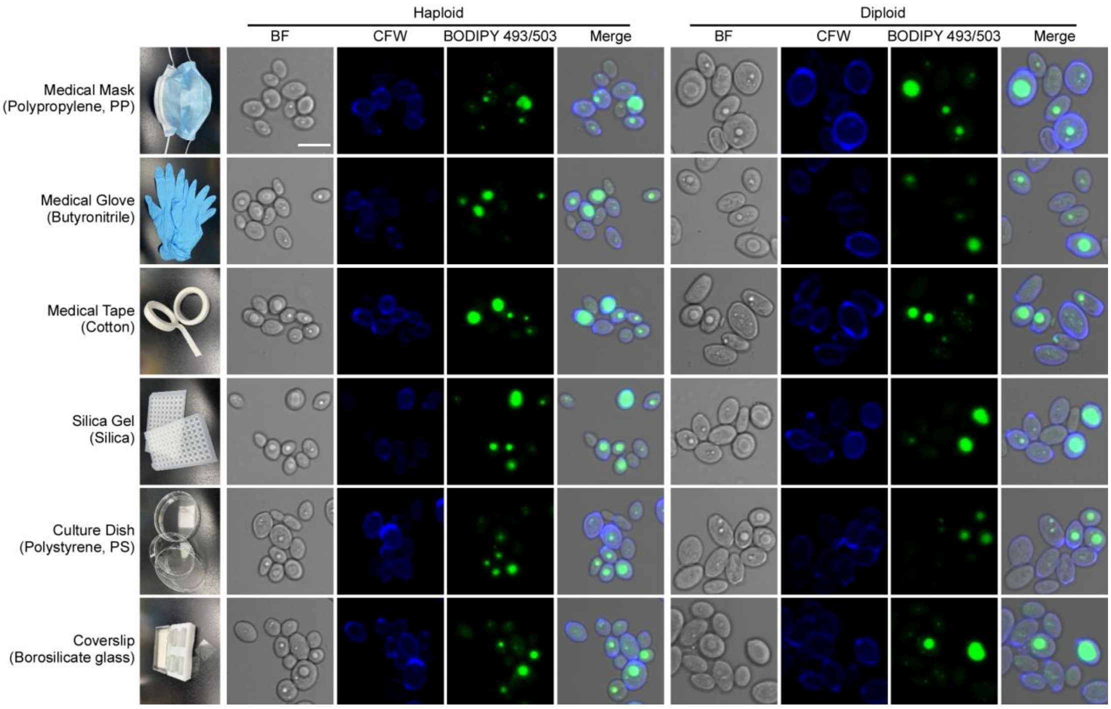

Supplementary Figure 4. Formation of gLDs in *C. auris* cells grown on the surfaces of medical supplies.

Haploid and diploid cells of strain AR0386 were initially grown on YPD medium for 2 days at 30°C, then 5 x 10^6 cells in 3 μL ddH_2O were then inoculated onto the surfaces of the medical supplies and incubated at 30°C for 14 days. *C. auris* cells were then collected, washed with PBS, and stained with BODIPY 493/503 and calcofluor white (CFW). BF, brightfield. Scale bar, 5 μm.

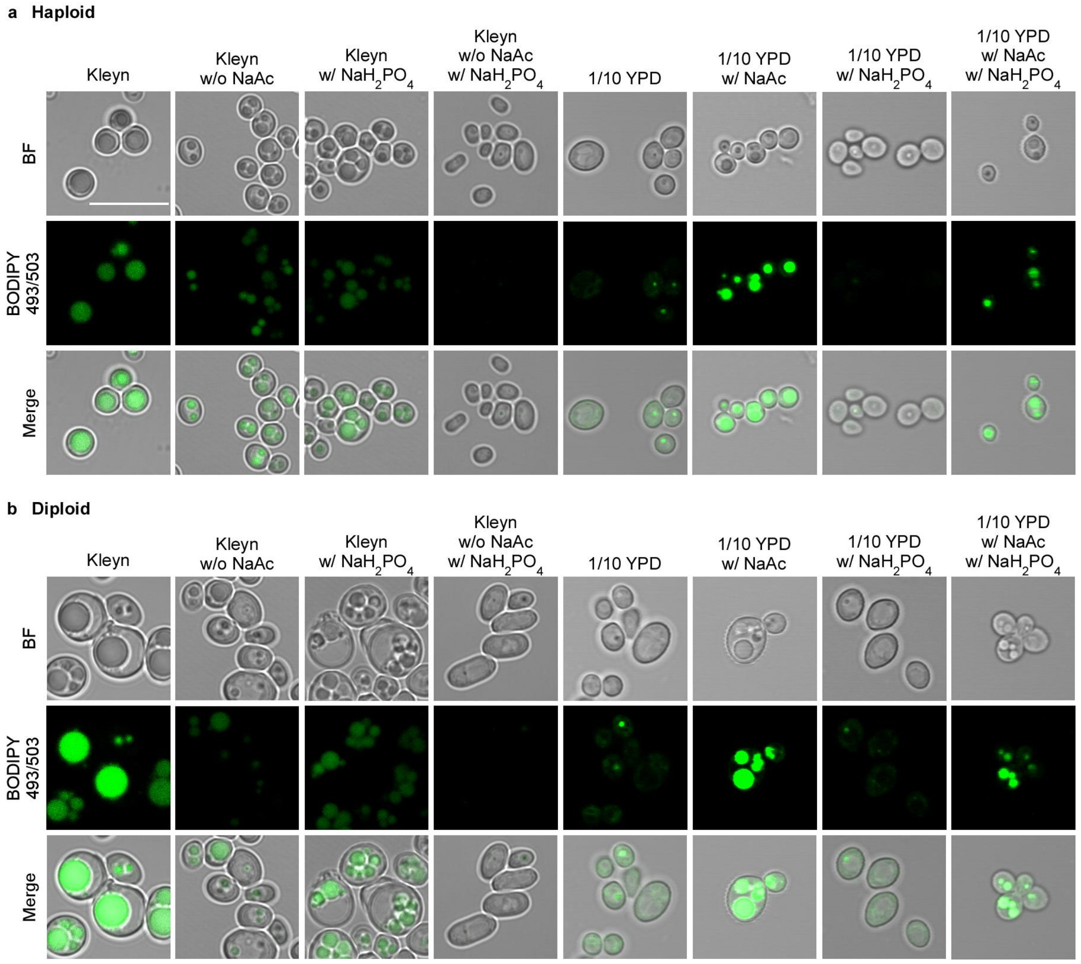

Supplementary Figure 5. Effect of acetate and phosphate (NaAc and NaH_2PO_4) on the formation of lipid droplets in *C. auris*.

Haploid (a) and diploid (b) cells were initially grown on YPD medium for 2 days at 30°C, and then spotted onto Kleyn and 1/10 YPD media at 5 x 10^6 cells in 10 µL ddH_2O and incubated at 30°C for 7 days. *C. auris* cells stained with BODIPY 493/503. BF, brightfield. Scale bar, 10 µm. Kleyn, Kleyn medium (original recipe, containing NaAc but no NaH_2PO_4). Modified Kleyn media: Kleyn w/o NaAc; Kleyn w/ NaH_2PO_4 (containing NaAc and NaH_2PO_4); Kleyn w/o NaAc w/ NaH_2PO_4 (mKleyn, containing NaH_2PO_4 but no NaAc). 1/10 YPD medium: 2% agar + 1:10 dilution of YPD nutrient solution. The presence and absence of NaH_2PO_4 and NaAc in modified YPD media are indicated in the figures and are the same as the modified Kleyn media.

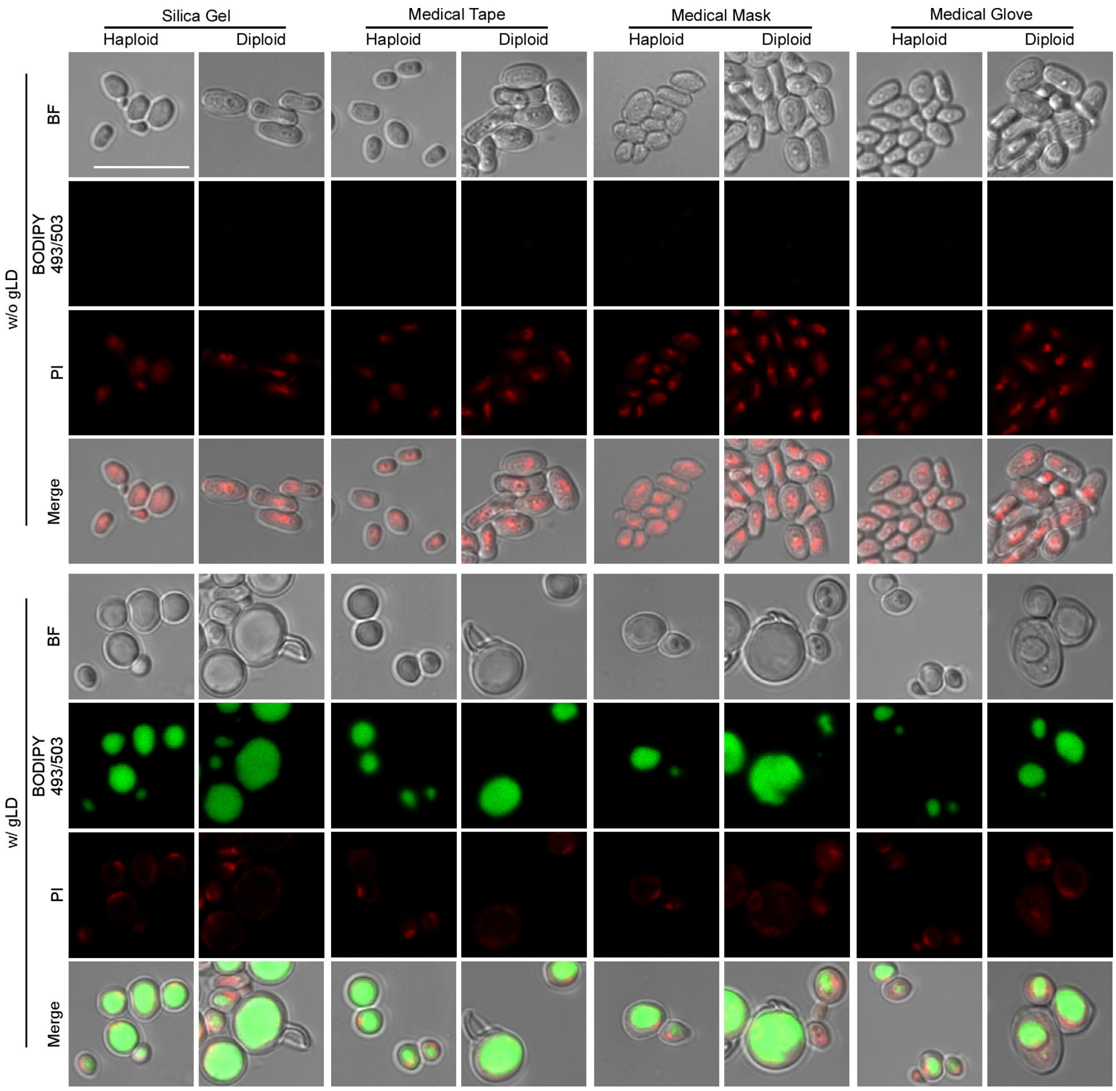

Supplementary Figure 6. Comparison of viability of *C. auris* cells with (w/) or without (w/o) gLDs on the surfaces of medical supplies.

C. auris cells w/ or w/o gLDs were collected from the cultures on the Kleyn or mKleyn media (w/o NaAc and w/ NaH_2PO_4) incubated at 30°C for 7 days, respectively. The cells (5×10^6 cells in 3 μL ddH_2O) were then spotted onto the surfaces of silica gel, medical tape, masks, or gloves at 30°C for 25 days. *C. auris* cells were collected, washed with PBS, and then stained with PI and BODIPY 493/503. BF, brightfield. Scale bar, 10 μm.

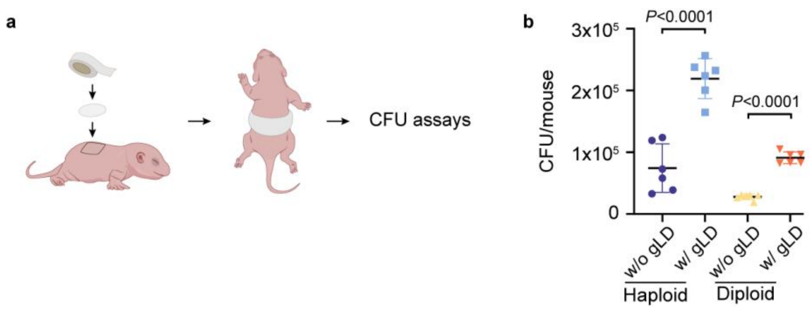

Supplementary Figure 7. Comparison of skin colonization of *C. auris* cells with (w/) or without (w/o) gLDs.

Haploid and diploid cells of strain AR0386 were examined. (a) Overview of experimental design. (b) CFU analysis of colonized *C. auris* cells. Approximately 2 x 10^6 *C. auris* cells w/ or w/o gLDs (in 2 μL PBS) were inoculated onto the dorsal back skin of newborn mice. After 3 days of infection, the infected skin areas were excised and used for CFU analyses on YPD medium. Six biological repeats were performed for each strain sample. Significant differences were determined using two-tailed, unpaired Student's *t*-tests. Dots represent the CFU numbers; error bars represent standard deviations.

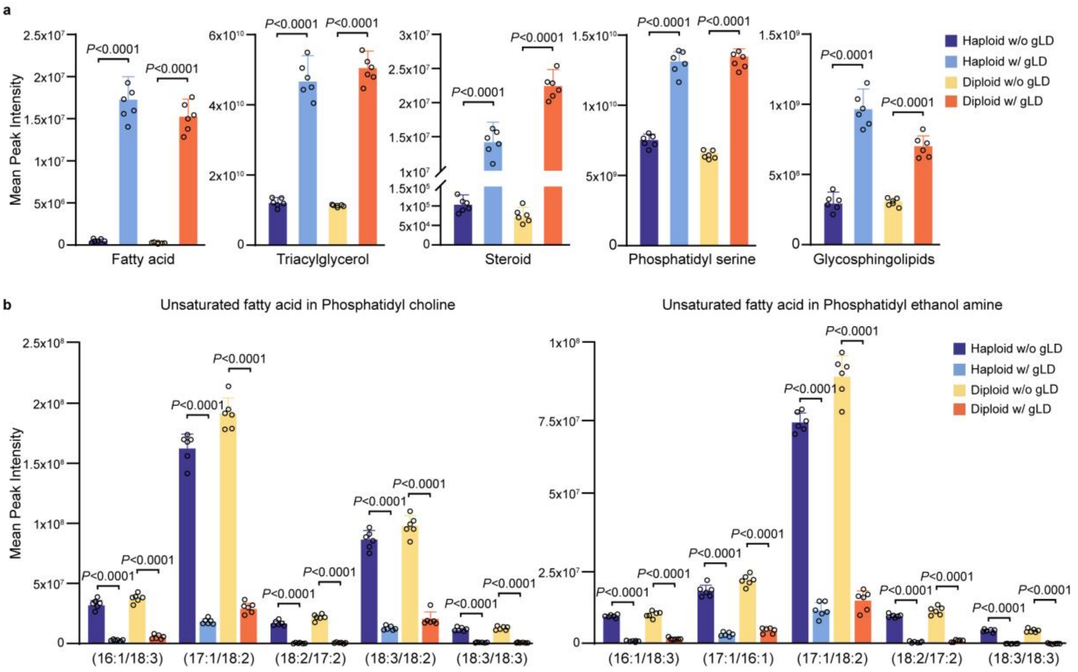

Supplementary Figure 8. Different levels of lipid components and unsaturated fatty acids in haploid and diploid *C. auris* cells with (w/) or without (w/o) gLDs.

(a) Relative levels of lipid components in haploid and diploid cells w/ or w/o gLDs. (b) The level of unsaturated fatty acids in phospholipids. The y-axis represents the mean peak intensity of lipids in each group. Significant differences were determined using two-tailed, unpaired Student's *t*-tests. Error bars represent standard deviations.

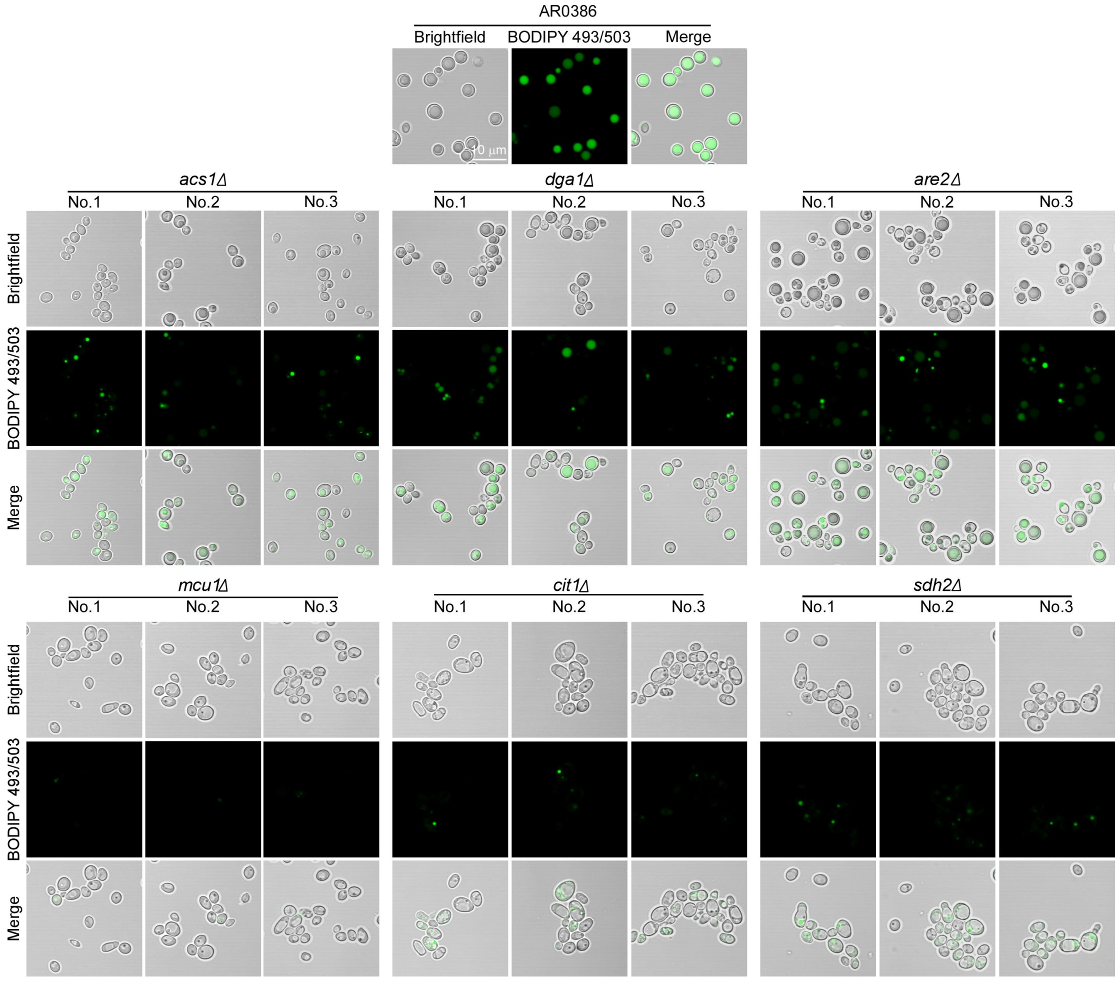

Supplementary Figure 9. Formation of gLDs in the WT and three independent isolates of the mutant strains.

C. auris cells of the WT strain AR0386, and the *acs1*Δ, *dga1*Δ, *are2*Δ, *mcu1*Δ, *cit1*Δ, and *sdh2*Δ mutant strains were initially grown on YPD medium at 30°C for 2 days, then replated onto Kleyn medium and incubated at 30°C for 7 days. Three independent isolates of each mutant strain were examined. BODIPY 493/503 staining assays were performed. Scale bar, 10 μm.

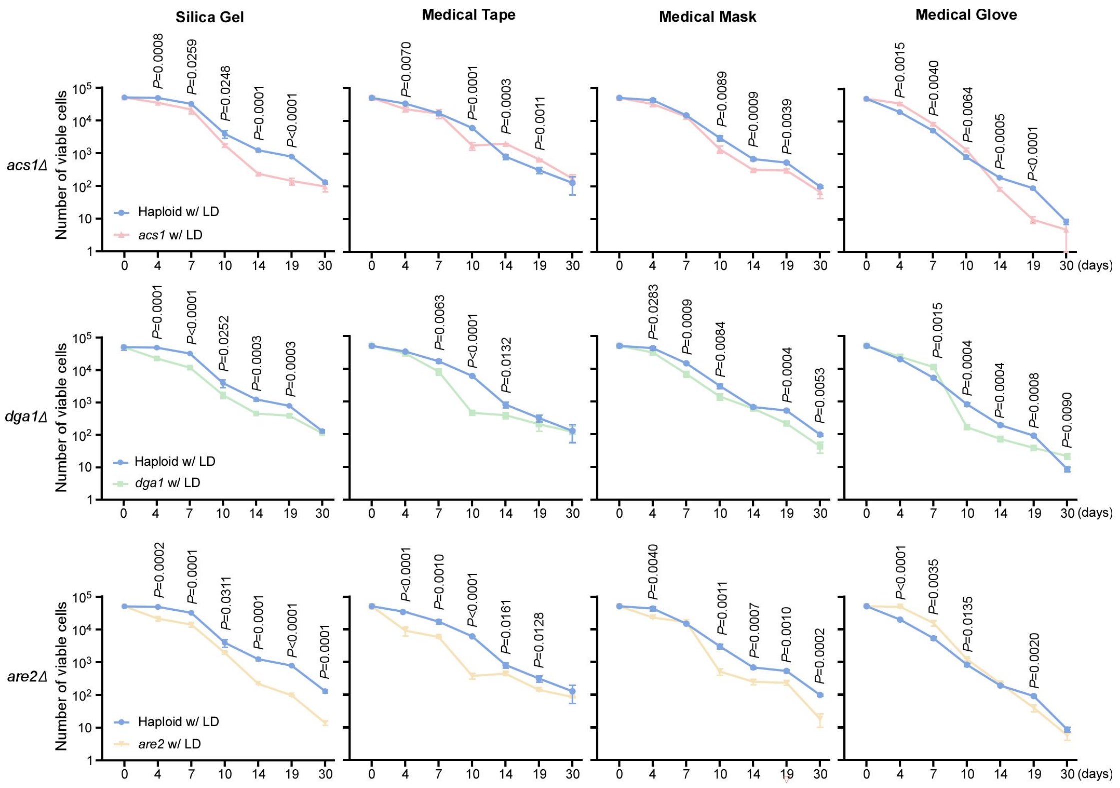

Supplementary Figure 10. Survival curves of the WT, and the *acs1Δ*, *dga1Δ*, and *are2Δ* mutant strains on the surfaces of silica gel, medical tape, masks, and gloves.

WT: blue curves. *C. auris* cells were first grown on YPD solid plates at 30°C for 2 days. Then 5 x 10^6 cells in 10 µL ddH_2O were spotted onto Kleyn medium and incubated at 30°C for 7days for the formation of lipid droplets. *C. auris* cells of the WT and mutant strains were collected. Approximately 5 x 10^6 cells in 3 µL ddH_2O of each strain were spotted onto the surface of silica gel, medical tape, masks, and gloves for viability analyses. CFU assays were performed on day 4, 7, 10, 14, 19, and 30. A logarithmic scale was used for the y-axis. Three replicates were performed. Significant differences were determined using two-tailed, unpaired Student's *t*-tests. Error bars represent standard deviations.

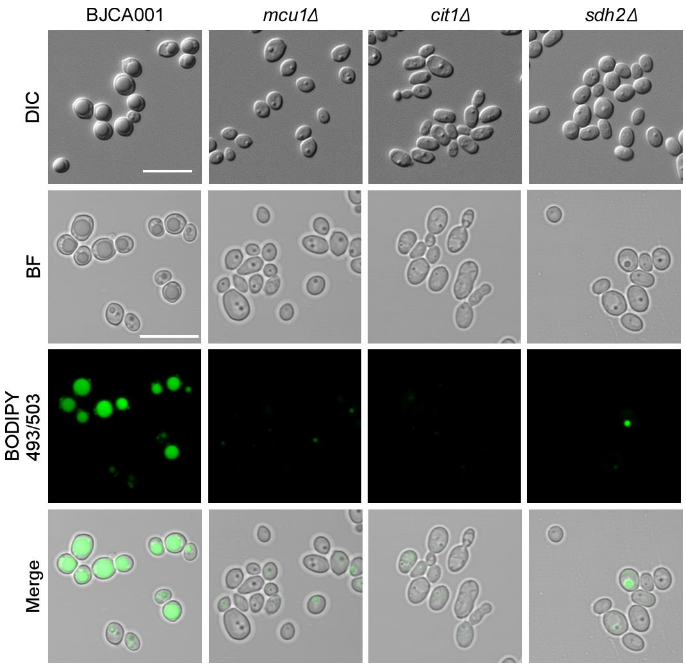

Supplementary Figure 11. Analysis of lipid droplet formation of the WT (BJCA001), *mcu1*Δ, *cit1*Δ, and *sdh2*Δ mutant strains on Kleyn medium. Haploid cells of the WT strain BJCA001 and the *mcu1*Δ, *cit1*Δ, and *sdh2*Δ mutant strains were initially grown on YPD medium for 2 days at 30°C. Approximately 5 x 10^6 cells in 10 μL ddH_2O were spotted onto Kleyn medium and incubated at 30°C for 7 days for the formation of lipid droplets. *C. auris* cells were stained with BODIPY 493/503. BF, brightfield. Scale bar, 10 μm.

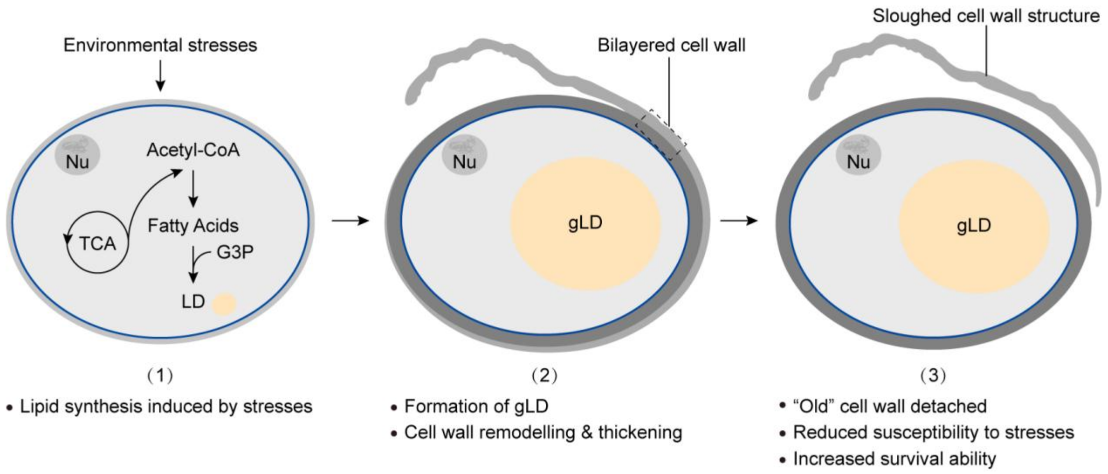

Supplementary Figure 12. Schematic diagram of a proposed gLD formation mechanism. Lipid droplets have crucial cross-functions in energy storage and stress response when *C. auris* cells encounter harsh environments. 1) After receiving environmental stress signals (such as NaAc, or other nutrient-poor conditions), TCA cycle or acetyl-CoA synthetase begins to continuously synthesize acetyl-CoA, the important precursor for lipid droplet formation. Fatty acids in the activated acyl-CoA form sequentially add to the backbone of glycerol-3-phosphate (G3P), and the intermediate products are catalyzed by several acyltransferases (including Dga1), then TAGs are synthesized. The structure of lipid droplets begins to take shape. 2) Lipid droplets enlarge with the continuous synthesis of TAGs, meanwhile cell wall remodeling or generation associated genes are highly expressed. The cell wall doubles in thickness and a distinct cell wall shedding phenomenon is observed, especially in diploid *C. auris* cells. 3) Due to gLD formation and cell wall thickening, *C. auris* cells show increased survivability and enhanced resistance to environmental stressors.

Supplementary Table 1. Relative composition of cell wall components of *C. auris* cells with or without giant lipid droplets.

Strains (AR0386)	Alkaline soluble	Alkaline insoluble	
	Glycoproteins (μg)	Chitins (μg)	β-glucans (μg)
Haploid w/o gLD	82.16 ± 0.21	55.45 ± 11.34	132.65 ± 32.97
Haploid w/ gLD	68.56 ± 2.13	134.01 ± 4.58	314.67 ± 57.57
Diploid w/o gLD	194.56 ± 10.03	114.94 ± 0.54	484.55 ± 45.11
Diploid w/ gLD	120.38 ± 1.89	363.85 ± 85.92	1039.18 ± 60.52

Notes: The relative contents of cell wall components (2 × 10^9 cells) were determined.
