# Supplementary material for: Candida auris cells form giant lipid droplets to survive in harsh environments
Source: Commun Biol. 2025 May 22;8:783. doi: 10.1038/s42003-025-08204-7 (PMC12098800; doi:10.1038/s42003-025-08204-7)
Supplement: Supplementary file 2 — Description of Additional Supplementary Files [file 42003_2025_8204_MOESM2_ESM.pdf]

Description of Additional Supplementary Files

File Name: Supplementary Data 1
Description: Lipidomic analysis of Candida auris cells with (w/) and without (w/o) gLDs.

File Name: Supplementary Data 2
Description: RNA-seq analysis of Candida auris cells with (w/) and without (w/o) gLDs.

File Name: Supplementary Data 3
Description: Primers used in this study.

File Name: Supplementary Data 4
Description: Strains used in this study.

File Name: Supplementary Data 5
Description: Source data.
